# Establishment of reference intervals for 41 serum biochemical analytes in healthy Chinese children

**DOI:** 10.1016/j.plabm.2026.e00537

**Published:** 2026-05-07

**Authors:** Qinlan Liu, Xiufa Zhang, Ling Zhuang, Yidan Wang, Xian Chen, Feng Wang

**Affiliations:** Clinical Laboratory, Shenzhen Baoan Women's and Children's Hospital, No.56, Yulv Road, Bao'an District, Shenzhen, 518100, China

**Keywords:** Reference interval, Pediatric, Biochemistry, Nonparametric method, Estimated glomerular filtration rate

## Abstract

**Purpose:**

Owing to the absence of appropriate pediatric reference intervals (RIs), this study aimed to establish age-specific RIs for serum biochemical analytes in healthy Chinese children.

**Methods:**

A total of 2627 residual serum samples were collected from children aged 28 days to 17 years at Shenzhen Baoan Women's and Children's Hospital. Forty-one analytes, including enzymes, proteins, lipids, electrolytes, and others, were measured using the Mindray BS-2800M analyzer. Outlier removal and RIs establishment were performed according to Clinical and Laboratory Standards Institute (CLSI) EP28 guideline. Age-dependent trajectories were visualized, and RIs were compared with multicenter studies. Additionally, the pediatric patterns of estimated glomerular filtration rate (eGFR) were also evaluated by equations.

**Results:**

Twenty-one analytes showed distinct age-dependent dynamics, including liver enzymes, prealbumin, bilirubin, immunoglobulins, renal biomarkers, cardiac enzymes, ferritin, and anti-streptolysin O. Meanwhile, electrolytes, proteins, glucose, and other anemia markers remained stable across ages. RIs for thirty-six analytes were largely consistent with comparative studies, although quantitative discrepancies were noted for triglyceride and anti-streptolysin O. In 806 children, eGFR increased rapidly during the first two years of life, with inter-formula variability. The European Kidney Function Consortium equation produced distribution pattern closest to measured ranges from the comparative study.

**Conclusions:**

This study established comprehensive pediatric biochemical RIs for forty-one serum analytes, with twenty-one exhibiting distinct age-dependent dynamics. Comparisons with landmark studies showed good agreement for thirty-six analytes. The eGFR equation selection requires caution, particularly in young children. These RIs provide reliable evidence to support cross-regional harmonization and help reduce inappropriate use of references in children.

## 1 Introduction

Reference intervals (RIs) are central to the accurate interpretation of laboratory results and pivotally inform clinical decision-making. Relying on inappropriate RIs could misclassify individuals as diseased or healthy, potentially leading to missed diagnoses, unnecessary follow-ups, or incorrect treatments. Thus, establishing reliable RIs is a fundamental responsibility of clinical laboratories. The Clinical and Laboratory Standards Institute (CLSI) guideline EP28-A3c remains the most widely adopted framework [1], providing a standardized process for defining, establishing, and verifying RIs. While the concept of an RI is straightforward, constructing intervals that are representative—considering age, sex, and regional differences—is truly demanding. A critical gap persists in pediatric-specific RIs, leaving most clinical laboratories to interpret children's biochemical test results against intervals derived from adult populations [2,3]. This practice is problematic because analyte concentrations in children change substantially with age and developmental stage, and using adult-based RIs carries a significant risk of misdiagnosis. Several major initiatives have sought to close this gap. The CALIPER (Canadian Laboratory Initiative on Pediatric Reference Intervals) project has prospectively established a comprehensive database of age- and sex-specific RIs for laboratory biomarkers in Canadian children [4], setting a benchmark for pediatric RIs research worldwide. In China, the PRINCE (Pediatric Reference Intervals in China) study has provided authoritative RIs for 14 biochemical markers based on a large and multicenter cohort of children, offering a valuable reference for the Chinese pediatric population [5]. Despite these contributions, the coverage of biochemical analytes remains limited [6].

The need for reliable pediatric RIs is therefore pressing. Moreover, to date no pediatric RIs have been fully reported for the Mindray (Shenzhen, China) system. Recognizing this gap, the present study aimed to establish comprehensive pediatric RIs for a wide panel of serum biochemical analytes using the BS-2800M chemistry analyzer, thereby providing reference data to support more accurate clinical diagnosis and health assessment in Chinese pediatric population.

## 2 Method and materials

### 2.1 Sample collection

Residual serum samples were collected from March 2023 to June 2024 at the Clinical Laboratory of Shenzhen Baoan Women's and Children's Hospital. The samples came from healthy children aged 28 days to 17 years who were undergoing routine physical examinations.

We applied the following inclusion criteria: (1) apparently healthy children aged 28 days to 17 years with normal physical examination findings; (2) no medication uses within the previous two weeks; (3) no history of cardiac disease, chronic illness, kidney injury, inherited metabolic disorders, autoimmune disease, or malignant tumor. Exclusion criteria were: (1) structural or morphological abnormalities of the liver, spleen, or kidneys on abdominal ultrasound; (2) diagnosed congenital diseases; (3) fever or acute severe illness within two weeks; (4) major surgery within the past month; (5) history of blood transfusion or severe internal bleeding.

A stratified sample collection strategy based on key developmental stages was adopted to cover children of all age groups as much as possible. Before sample collection, the target collected pediatric population was prospectively divided into seven pre-defined age groups according to growth and development phases recognized. These groups were: infancy (breastfeeding period: 28 days-<6 months; weaning period: 6 months-<1 year), toddlerhood (1-<3 years), preschool age (3-<6 years), school age (6-<9 years), pre-adolescence (9-<12 years), and adolescence (12-<18 years). The final age partitioning of the RIs depended on the distribution characteristics and statistical differences of each analyte.

### 2.2 Analyte selection

A total of 41 serum test analytes were selected for the establishment of pediatric RIs, including liver function analytes [Aspartate aminotransferase (AST), Alanine aminotransferase (ALT), Alkaline phosphatase (ALP), γ-glutamyl-transferase (γ-GT), Total protein (TP), Albumin (Alb), Prealbumin (PA), Total bilirubin (TBIL), and Direct bilirubin (DBIL)], renal function analytes [Creatinine (Crea), Urea (Urea), Uric acid (UA) and Cystatin C (CysC)], lipid tests [Total cholesterol (TC), Triglycerides (TG), High Density Lipoprotein-Cholesterol (HDL-C), and Low Density Lipoprotein-Cholesterol (LDL-C)], cardiovascular analytes [Creatine kinase (CK), Creatine kinase MB isoenzyme (CK-MB), Lactate dehydrogenase (LDH), α-Hydroxybutyrate dehydrogenase (α-HBDH), and Myoglobin (MYO)], ion analytes [Potassium (K), Sodium (Na), Chloride (Cl), Calcium (Ca), Magnesium (Mg), Phosphate (P), Carbon dioxide (CO_2_)], specific protein analytes [Immunoglobulin G (IgG), Immunoglobulin M (IgM), Immunoglobulin A (IgA), and Immunoglobulin E (IgE)], glucose [glucose oxidase method (Glu-GOD) and hexokinase method (Glu-HK)], anemia analytes [Iron (Fe), Ferritin (FER), Transferrin (TRF), Unsaturated iron-binding capacity (UIBC) and Total iron-binding capacity (TIBC)], and rheumatism analyte [Antistreptolysin O (ASO)]. The collection, processing, and storage of serum samples were performed according to the specifications given in document H18-A3 [7].

The reagents for those analytes were all from Mindray (Shenzhen, China), except for TIBC, which was a calculated parameter. Once serum Fe and UIBC values are determined, TIBC is calculated using a straightforward formula: TIBC = Fe + UIBC.

### 2.3 Sample testing process

All samples were collected and tested on the same day to ensure freshness, and testing was completed within 2 h after collection using the Mindray BS-2800M chemistry analyzer and paired reagents at the Clinical Laboratory of Shenzhen Baoan Women's and Children's Hospital. After testing, samples were refrigerated for three days. The calibration process and sample testing were conducted according to the manufacturer's instructions. As for traceability, biochemical analytes with reference materials were traced to reference materials, and those without reference materials were traced to reference methods. The abbreviations, approaches and traceability for biochemical analytes used in this study were listed in Supplementary Table 1 (Table S1). The information of calibrator and control was listed in Table S2.

### 2.4 Quality control

The samples with hemolysis, jaundice, and hyperlipidemia were excluded. Sample analysis was conducted at the Clinical Laboratory of Shenzhen Baoan Women's and Children's Hospital. The laboratory passed the on-site assessment by China National Accreditation Service (CNAS) in 2018 and the ISO15189 Medical Laboratory Accreditation in 2021. The used chemistry analyzer was preventively maintained by the manufacturer to ensure that the performance of the detection system meets the requirements, including precision, accuracy, analytical measurement range, carryover contamination, etc. [8,9].

### 2.5 Data processing and reference intervals establishment

The process was conducted according to the EP28-A3c [1] guideline issued by the clinical laboratory standard institute (CLSI). Data cleaning was performed before analysis (The cleaning subjects included unreasonable data with abnormal negative value or wrong-input, and data lacking any age or gender). Reference value distributions were plotted by age, and separated by sex for each parameter. The normality of the measured values of each analyte was determined by Kolmogorov-Smirnov test. Outliers were initially screened in the overall population for each analyte. For normally distributed and skew-normally distributed data, outliers were eliminated by Tukey method. For skew distribution data, outliers were eliminated by Dixon method. Given age-related physiological changes, initial identified outliers were supplemented by visual verification based on distribution scatter plots to ensure that the overall screening did not inadvertently mask outliers within specific age changes. Then, scatter plots were visually inspected to identify potential outliers that remained clearly dissociated from the main cluster of each pre-defined age group. The identified outliers were statistically confirmed and removed. This combined approach minimized subjective elimination errors in pediatric population.

Age-specific partitioning was determined using a two-step approach. First, scatter plots of testing data against age, stratified by sex, were visually inspected to identify tendency and inflection points of each parameter. If the measured values of the analyte were observed a trending vary significantly with age or gender through visual inspection, it was preliminarily considered to conduct age/gender partitioning. For varying parameter in which the trend and inflection points were found to be different from the pre-defined physiological development groups, re-grouping evaluation was performed for further analysis. Then, the Harris-Boyd method was applied to determine the necessity of age partitioning. For each pair of adjacent age groups, z values were calculated by standard deviation (s), mean (x), and sample size (n). And a critical value (z∗) was calculated based on sample sizes of adjacent two groups. The Harris Boyd equation was shown in Fig. S1. If calculated z-value exceeding critical z-value (z > z∗), it indicated a statistically significant difference between the two groups and partition was recommended. If the test was not significant (z ≤ z∗), the groups were considered candidates for merging. Additionally, if the larger standard deviations in one subgroup exceeds 1.5 times of the smaller one in next subgroup, group partition was also advised. After that, One-way ANOVA with Student-Newman-Keuls (SNK) post-hoc test was performed as complementary verification to confirm that the final partitioned groups were statistically distinct. For gender partitioning, the Mann-Whitney *U* test was used to analyze statistical differences between males and females within each final age group.

According to the guidance of EP28-A3c, the non-parametric method was employed to estimate the reference limits of each parameter for subgroups with sample size of at least 120. For subgroups with sample size below 120, the Box–Cox transformation was applied to approximate normality, with the optimal transformation parameter λ estimated by maximum likelihood. Subsequently, the robust method was used to calculate the reference limits. The unilaterality or bilateral of the RI was determined by the clinical decision threshold of each analyte. For analytes suggestive of disease at both abnormal elevation and decrease, two-sided RI (2.5th and 97.5th percentiles) was established. For analytes suggestive of disease only when abnormally elevation or decrease, one-sided RI (5th or 95th percentiles) was established. The 90% confidence intervals (CIs)were calculated for both reference limits. Statistical analyses were performed using Medcalc (version 22, Ostend, Belgium. https://www.medcalc.org) and Graphpad Prism (version 10.1.2, San Diego, CA, USA. www.graphpad.com).

### 2.6 Evaluation of pediatric estimated glomerular filtration rate

To evaluate the age-dependent trajectories of estimated glomerular filtration rate (eGFR) across different developmental stages, seven reported equations were respectively used to calculate eGFR, including the Chronic Kidney Disease Epidemiology Collaboration (CKD-EPI) Crea-CysC [10,11], Schwartz Bedside [12], European Kidney Function Consortium (EKFC) [13], Chronic Kidney Disease in Children (CKiD) U25 [14], Counahan-Barratt formula [15], Caucasian and Asian Pediatric and Adult (CAPA) [16], Full-age Spectrum (FAS) [17,18], as shown in Table S3. Scatter plots with Locally Weighted Scatterplot Smoothing (LOWESS) regression were utilized to capture the non-linear physiological trends of eGFR calculated by the three equations across ages, stratified by gender. Violin plots and Kernel Density Estimation (KDE) were utilized to evaluate the overall data distribution and central tendencies of the results, providing a comprehensive visualization of the probability density, median, interquartile range (IQR) and potential estimation. All graphical visualizations and statistical plots were generated using Python (with matplotlib and seaborn libraries).

## 3 Results

### 3.1 General information of reference population

**Fig. 1:**
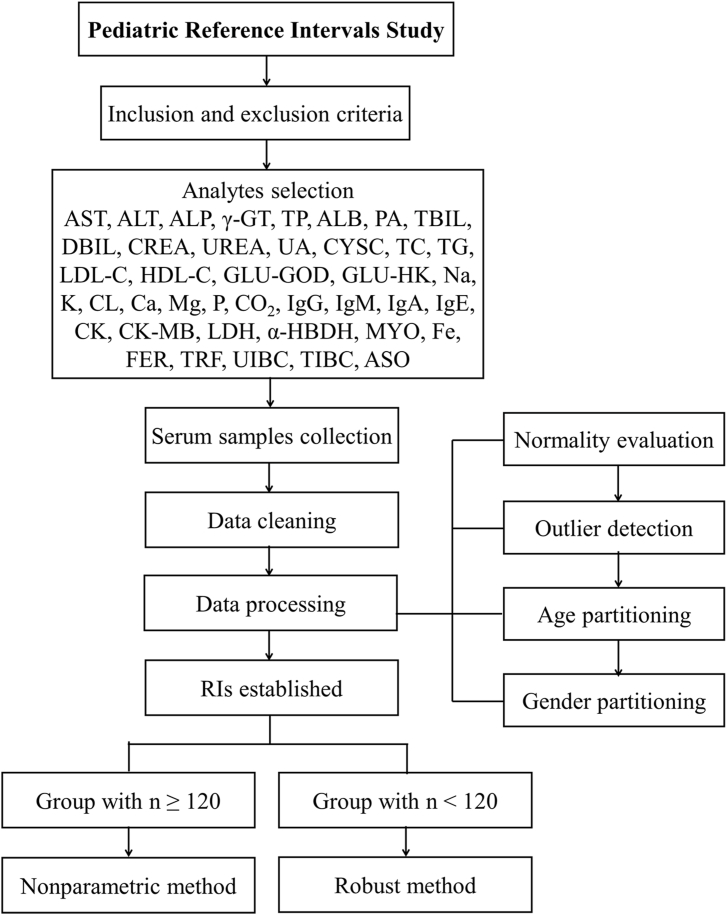
The flowchart for establishing RIs in this study.

The analysis process of this study was described in Fig. 1. A total of 2627 children's serum samples were included to establish RIs according to inclusion and exclusion criteria, comprising 1453 males and 1174 females. The sample size classified by pre-defined seven groups were shown in Table S4. Owing to the blood volume of the residual samples in the same tube was limited to meet the needs of detecting all analyses at the same time, the samples were tested in three batches, respectively. The sample size and analytes tested for each batch were detailed in Table S5. Daily internal quality control was performed throughout all batches, and results were accepted when quality control values were within specification. The original sample size corresponding to each analyte was shown in Table S6.

### 3.2 Distribution pattern of serum biochemical RIs

**Fig. 2:**
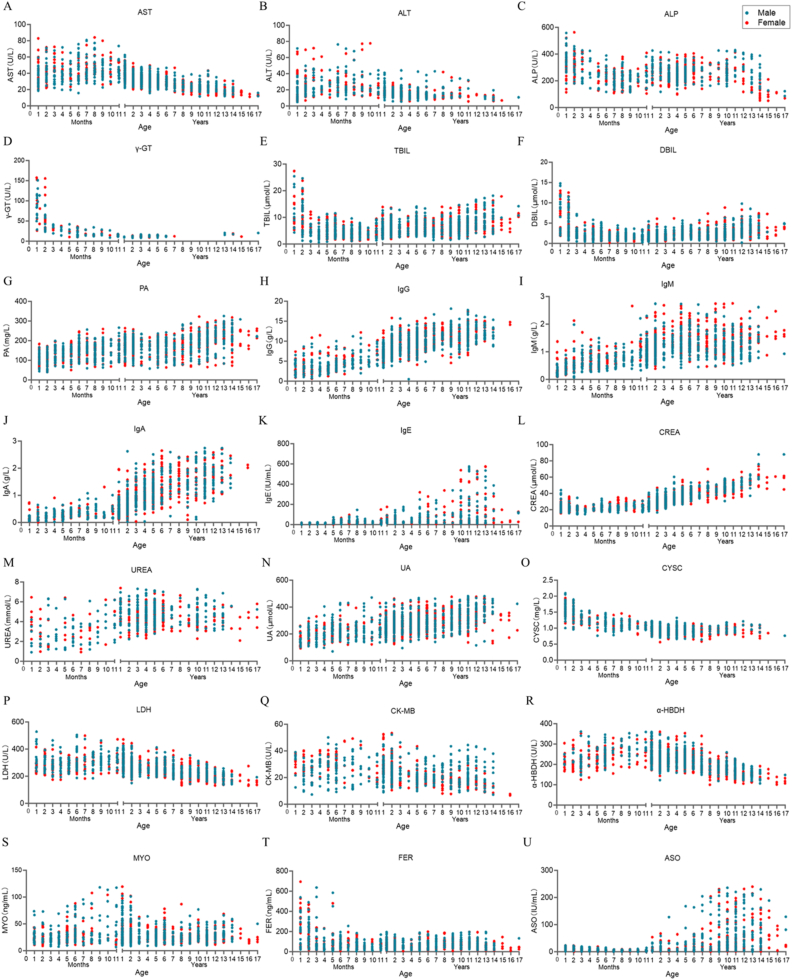
Age-dependent **concentration distribution plots of serum biochemical analytes related to age changes.** (A-U). The scatter plots of 21 biochemical markers concentrations with age and gender in the present study. The blue color represented the values of males and the red for the females. The distribution plots of biochemical markers in the present study were included all individuals aged from 28d to <18y. The 28-day-old infants were drawn in the 1-month-old group.

As summarized in Table 1, RIs were established for 41 serum biochemical analytes. Among those, 21 analytes revealed distinct age-dependent dynamics, including liver parameters (AST, ALT, ALP, γ-GT, PA, TBIL, DBIL), kidney parameters (Crea, Urea, UA, CysC), cardiovascular parameters (LDH, CK-MB, α-HBDH, MYO), immunoglobulins (IgA, IgG, IgE, IgM) and others (FER, ASO). Other indicators, such as ions (Ca, Mg, P, Na, K, Cl), proteins (Alb, TP), glucose and anemia parameters (Fe, TRF, UIBC, TIBC), showed relatively stable changes. The distribution plots of analytes with age-dependent changes were shown in Fig. 2.

**Table 1:** RIs of the serum clinical chemistry analytes in apparently healthy check-up children of Shenzhen, China.

Analyte	Unit	Group	n	Mean	Median	LL	UL	90% CI for LL	90% CI for UL
**AST**	U/L	28 days-<1 year	261	42.15	40.67	22.29	75.70	20.48-25.37	70.77-80.76
		1-<2 years	120	39.94	39.61	24.48	55.68	22.03-28.47	53.31-62.33
		2-<7 years	801	31.34	30.94	21.91	44.81	20.65-22.82	43.32-45.61
		7-<13 years	228	23.56	22.85	13.30	38.58	11.91-15.77	36.24-43.19
		13-<18 years	Male 27 Female 32	Male 17.98 Female 16.68	Male 17.94 Female 16.22	Male 11.21 Female 10.33	Male 33.44 Female 27.44	Male 10.19-12.49 Female 9.40-11.38	Male 26.53-42.53 Female 23.92-31.43
**ALT**	U/L	28 days-<1 year	240	26.93	23.02	8.38	70.94	6.91-9.52	64.88-77.05
		1-<7 years	370	15.42	14.25	8.42	30.91	6.60-9.05	26.10-38.62
		7-<18 years	Male 32 Female 45	Male 16.44 Female 12.75	Male 12.69 Female 11.69	Male 7.15 Female 6.25	Male 46.55 Female 28.18	Male 6.33-8.40 Female 5.59-7.11	Male 28.04-68.45 Female 21.68-37.26
**AST/ALT**	/	28 days-<1 year	247	1.78	1.73	0.79	2.97	0.54-0.84	2.90-3.05
		1-<7 years	365	2.25	2.27	1.02	3.30	0.79-1.32	3.25-3.40
		7-<18 years	76	1.69	1.70	0.57	2.76	0.38-0.77	2.60-2.90
**ALP**	U/L	28 days-<6 months	122	308.79	312.50	135.47	517.64	113.55-143.52	457.83-562.10
		6 months-<1 year	167	213.21	210.90	118.61	361.88	111.70-124.48	312.08-388.54
		1-<7 years	344	254.43	252.83	157.74	379.32	117.28-167.34	369.35-408.47
		7-<11 years	71	266.32	272.72	153.48	396.12	138.29-172.18	373.07-418.16
		11-<15 years	Male 41 Female 30	Male 292.87 Female 161.79	Male 303.06 Female 147.92	Male 122.97 Female 77.39	Male 530.94 Female 384.43	Male 94.92-153.01 Female 69.81-88.20	Male 472.18-587.43 Female 284.10-511.33
		15-<18 years	14	98.77	96.32	37.71	238.28	28.97-55.42	168.55-332.30
**γ-GT**	U/L	28 days-<6 months	71	50.43	49.51	13.45	159.03	10.72-16.58	134.41-184.86
		6 months-<18 years	123	15.43	13.27	8.64	35.41	7.90-9.27	33.37-35.84
**TP**	g/L	28 days-<6 months	145	58.54	58.47	47.00	70.00	46.09-49.14	67.6-76.5
		6 months-<1 year	156	64.28	63.89	54.90	74.30	51.16-57.57	73.43-75.16
		1-<6 years	528	67.47	67.46	59.60	75.30	58.84-60.39	73.99-76.57
		6-<18 years	234	70.55	70.47	61.90	80.70	56.06-63.75	78.83-82.15
**Alb**	g/L	28 days-<6 months	141	39.59	39.89	30.85	47.63	30.35-31.76	46.00-48.29
		6 months-<18 years	663	44.85	45.02	39.45	49.22	37.98-40.46	48.90-49.98
**PA**	mg/L	28 days-<6 months	288	120.50	120.28	50.40	190.53	43.44-58.57	179.48-196.83
		6 months < 8 years	748	161.39	167.92	78.31	233.90	74.94-83.78	229.45-238.41
		8-<18 years	372	202.51	210.42	79.99	293.46	74.34-88.75	287.67-304.25
**TBIL**	μmol/L	28 days-<3 months	87	11.79	12.94	2.04	30.40	1.39-2.98	27.22-33.16
		3 months-<1 year	380	4.65	4.40	1.38	9.96	1.08-1.74	9.45-11.11
		1-<12 years	698	6.16	5.74	2.42	12.75	2.18-2.68	11.81-13.30
		12 -<18 years	131	9.10	8.35	3.82	17.20	2.63-41.7	16.07-18.09
**DBIL**	μmol/L	28 days-<3 months	136	7.41	7.35	1.30	15.63	0.74-1.85	14.50-15.98
		3 months-<7 months	190	2.12	1.87	0.54	5.15	0.44-0.78	4.31-6.86
		7 months-<1 years	202	1.58	1.54	0.45	3.40	0.21-0.57	2.86-3.76
		1 -<10 years	570	2.05	1.82	0.73	4.52	0.57-0.79	4.21-5.28
		10 -<18 years	273	3.17	2.72	1.00	7.11	0.82-1.20	5.51-8.50
**Crea**	μmol/L	28 days-<2 years	471	23.51	23.01	16.88	34.54	16.20-17.46	32.74-36.07
		2-<4 years	290	30.5	30.14	21.74	42.19	19.38-22.81	38.81-44.52
		4-<12 years	555	38.6	37.28	27.83	54.36	27.44-28.58	53.21-56.37
		12-<18 years	Male 30 Female 33	Male 58.52 Female 56.29	Male 57.88 Female 57.64	Male 39.77 Female 35.24	Male 93.51 Female 77.70	Male 36.79-43.93 Female 30.28-41.33	Male 79.76-110.67 Female 72.82-82.49
**Urea**	mmol/L	28 days-<6 months	133	2.64	2.44	0.99	5.32	0.91-1.12	4.91-5.66
		6 months-<1 year	174	3.08	3.00	1.13	5.92	0.78-1.40	5.07-6.21
		1-<18 years	Male 235 Female 249	Male 4.80 Female 4.36	Male 4.73 Female 4.31	Male 2.85 Female 2.37	Male 7.03 Female 6.36	Male 2.23-3.13 Female 2.09-2.69	Male 6.66-7.28 Female 6.14-6.85
**UA**	μmol/L	28 days-<1 year	580	209.18	203.26	99.91	386.35	94.60-108.14	351.13-412.86
		1-<11 years	777	267.12	265.81	159.96	368.38	152.36-173.54	363.02-370.84
		11-<18 years	Male 101 Female 77	Male 366.97 Female 284.72	Male 367.90 Female 291.46	Male 194.76 Female 123.90	Male 480.95 Female 395.65	Male 157.56-227.75 Female 88.72-158.87	Male 467.13-493.52 Female 382.05-407.47
**CysC**	mg/L	28 days-<5 months	128	1.44	1.42	1	1.9	0.96-1.09	1.85-2.09
		5 months-<1 year	136	1.13	1.13	0.81	1.44	0.76-0.91	1.36-1.50
		1-<18 years	Male 342 Female 282	Male 0.94 Female 0.93	Male 0.90 Female 0.90	Male 0.72 Female 0.69	Male 1.18 Female 1.10	Male 0.70-0.74 Female 0.65-0.75	Male 1.16-1.21 Female 1.08-1.15
**TC**	mmol/L	28 days-<2 years	658	3.73	3.62	2.30	5.58	2.16-2.42	5.36-5.80
		2-<10 years	664	4.11	4.07	2.63	5.78	2.43-2.69	5.59-6.14
		10-<18 years	263	3.79	3.79	2.49	5.16	2.42-2.71	4.87-5.69
**TG**	mmol/L	28 days-<2 years	681	1.22	1.09	0.44	2.54	0.41-0.47	2.43-2.71
		2-<10 years	727	0.84	0.73	0.31	2.08	0.29-0.34	1.85-2.23
		10-<18 years	260	0.89	0.79	0.35	1.85	0.32-0.41	1.66-2.02
**LDL-C**	mmol/L	28 days-<5 months	183	1.89	1.83	0.91	3.00	0.70-1.10	2.87-3.19
		5 months-<2 years	325	2.37	2.36	1.22	3.48	0.97-1.40	3.45-3.51
		2-<11 years	332	2.38	2.37	1.28	3.40	1.13-1.44	3.33-3.47
		11 -<18 years	149	2.16	2.18	1.28	3.27	0.83-1.36	3.13-3.40
**HDL-C**	mmol/L	28 days-<7 months	274	1.25	1.20	0.40	2.30	0.30-0.60	2.10-2.60
		7 months-<2 years	322	1.18	1.17	0.62	1.80	0.51-0.65	1.68-1.89
		2-<11 years	729	1.47	1.46	0.80	2.21	0.77-0.82	2.14-2.28
		11-<18 years	210	1.39	1.38	0.82	2.01	0.76-0.88	1.88-2.07
**Glu-HK**	mmol/L	28 days-<2 years	243	4.99	5.07	3.86	5.80	3.75-3.95	5.75-5.84
		2-<5 years	169	4.62	4.61	3.60	5.90	3.21-3.75	5.59-6.07
		5-<18 years	Male 121 Female 149	Male 4.83 Female 4.58	Male 4.86 Female 4.52	Male 3.55 Female 3.48	Male 6.00 Female 5.93	Male 3.53-3.62 Female 3.36-3.62	Male 5.84-6.06 Female 5.67-5.97
**Glu-GOD**	mmol/L	28 days-<2 years	243	5.03	5.12	3.86	5.78	3.73-3.99	5.76-5.92
		2-<5 years	169	4.69	4.66	3.63	6.02	3.25-3.80	5.65-6.20
		5-<18 years	Male 126 Female 149	Male 4.92 Female 4.65	Male 4.95 Female 4.59	Male 3.59 Female 3.61	Male 6.09 Female 5.94	Male 3.53-3.67 Female 3.42-3.68	Male 5.98-6.17 Female 5.78-6.05
**Na**	mmol/L	28 days-<6 months	160	140.92	140.57	136.35	147.96	135.78-137.30	146.01-153.49
		6 months-<1 year	147	141.35	141.13	136.81	146.93	136.04-137.70	145.70-147.97
		1-<18 years	788	140.40	140.40	136.40	143.86	135.93-137.01	143.45-144.17
**K**	mmol/L	28 days-<1 year	172	5.06	4.99	3.90	5.90	3.62-4.29	5.83-6.04
		1-<7 years	535	4.47	4.47	3.80	5.20	3.68-3.90	5.12-5.27
		7-<18 years	158	4.38	4.38	3.70	5.00	3.47-3.88	4.90-5.36
**CL**	mmol/L	28 days-<6 months	308	107.69	107.68	103.00	114.00	102.06-103.23	112.05-114.27
		6 months-<18 years	788	106.28	106.30	102.00	111.00	101.15-102.40	110.22-110.97
**Ca**	mmol/L	28 days-<2 years	248	2.54	2.54	2.34	2.73	2.31-2.36	2.71-2.75
		2-<5 years	160	2.53	2.54	2.39	2.68	2.36-2.41	2.65-2.74
		5-<18 years	249	2.50	2.50	2.36	2.63	2.35-2.37	2.61-2.67
**Mg**	mmol/L	28 days-<4 months	132	0.93	0.90	0.80	1.07	0.80-0.80	1.00-1.10
		4 months-<2 years	306	0.96	1.00	0.80	1.10	0.80-0.80	1.00-1.10
		2-<18 years	856	0.90	0.90	0.80	1.00	0.80-0.80	1.00-1.00
**P**	mmol/L	28 days-<6 months	98	2.07	2.03	1.48	2.48	1.37-1.59	2.42-2.53
		6 months-<1 year	74	1.80	1.77	1.39	2.25	1.33-1.45	2.16-2.32
		1-<6 years	281	1.58	1.57	1.31	1.96	1.25-1.34	1.91-1.98
		6-<18 years	Male 58 Female 81	Male 1.59 Female 1.56	Male 1.60 Female 1.57	Male 1.19 Female 1.13	Male 1.98 Female 1.88	Male 1.12-1.27 Female 1.05-1.21	Male 1.91-2.05 Female 1.84-1.92
**CO_2_**	mmol/L	28 days-<2 years	135	16.69	16.57	11.15	22.51	10.39-12.55	20.47-23.48
		2-<18 years	147	19.80	19.89	15.83	23.20	14.18-16.83	22.42-23.73
**IgG**	g/L	28 days-<6 months	253	3.77	3.56	0.80	8.36	0.49-1.21	7.20-8.60
		6 months-<1 year	215	5.09	4.81	2.26	9.97	1.76-2.59	0.96-10.76
		1-<2 years	120	7.16	7.13	3.91	11.98	3.34-4.58	9.81-12.65
		2-<5 years	308	8.66	8.50	5.10	13.20	4.90-5.40	12.50-13.90
		5-<8 years	259	10.02	9.90	6.20	14.40	5.30-6.80	13.90-15.00
		8-<11 years	141	11.20	11.40	6.22	15.00	4.50-7.30	14.30-18.10
		11-<18 years	146	12.16	12.15	7.94	16.76	6.50-8.70	15.30-17.80
**IgM**	g/L	28 days-<4 months	142	0.40	0.30	0.10	1.40	0.10-0.10	1.00-2.10
		4 < 10 months	194	0.71	0.70	0.30	1.30	0.30-0.40	1.20-1.50
		10 months-<2 years	153	1.02	1.00	0.49	2.20	0.30-0.50	1.60-2.70
		2-<5 years	202	1.25	1.20	0.60	2.40	0.40-0.60	2.10-2.70
		5-<11 years	345	1.37	1.30	0.60	2.50	0.50-0.80	2.40-2.70
		11-<18 years	201	1.31	1.31	0.64	2.22	0.55-0.71	2.01-2.52
**IgA**	g/L	28 days-<4 months	156	0.18	0.16	0.01	0.43	0.00-0.31	0.38-0.47
		4 < 10 months	202	0.43	0.40	0.09	0.87	0.02-0.13	0.83-0.88
		10 months-<2 years	132	0.49	0.45	0.14	1.14	0.11-0.16	1.00-1.21
		2-<5 years	307	0.87	0.84	0.16	1.86	0.04-0.26	1.72-2.14
		5-<8 years	191	1.25	1.19	0.50	2.38	0.36-0.50	2.15-2.59
		8-<11 years	102	1.56	1.54	0.44	2.64	0.29-0.61	2.51-2.77
		11-<18 years	121	0.71	2.74	0.75	2.70	0.63-0.87	2.58-2.83
**IgE**	IU/mL	28 days-<5 months	155	9.07	8.83	≤16.38		15.51-17.52	
		5 months-<2 years	193	31.93	21.98	≤96.07		79.99-142.37	
		2-<5 years	46	30.99	29.91	≤147.67		97.31-219.21	
		5-<10 years	62	39.47	35.43	≤312.49		202.29-477.34	
		10-<14 years	142	151.03	49.93	≤535.38		517.60-560.01	
		14-<18 years	20	84.56	111.46	≤409.60		251.67-537.83	
**CK**	U/L	28 days-<4 months	138	113.39	104.28	26.20	235.39	21.94-43.21	218.96-248.59
		4 months-<2 years	328	127.12	125.90	41.13	248.37	35.50-48.60	235.20-252.20
		2-<18 years	705	113.68	106.50	17.86	227.84	35.70-46.40	216.90-237.80
**CK-MB**	U/L	28 days-<3 years	588	28.37	28.60	12.56	43.03	10.82-13.72	41.80-44.04
		3-<18 years	337	17.18	17.35	8.64	25.02	7.12-9.66	24.14-25.73
**LDH**	U/L	28 days-<3 years	797	287.70	289.72	196.16	468.09	182.78-208.50	436.83-498.89
		3-<8 years	247	257.61	254.31	193.49	335.66	184.82-197.19	322.24-346.25
		8-<12 years	216	219.51	220.01	144.68	294.75	142.41-155.73	289.58-301.69
		12-<18 years	123	183.09	179.68	131.57	241.08	116.13-136.55	236.49-248.74
**α-HBDH**	U/L	28 days-<2 years	627	236.36	233.26	159.24	324.44	151.59-165.23	320.87-328.97
		2-<8 years	531	221.09	220.08	163.57	282.72	158.73-166.82	278.00-287.39
		8-<12 years	209	176.13	174.50	121.17	225.20	117.74-126.74	220.67-226.76
		12-<18 years	120	140.57	139.86	100.64	180.80	99.02-106.20	173.84-183.50
**MYO**	ng/mL	28 days-<6 months	192	25.55	21.60	9.58	69.41	8.46-11.74	51.93-73.40
		6 months-<1 year	128	38.10	30.05	10.53	111.99	6.68-12.90	91.92-117.93
		1-<3 years	204	33.72	26.88	12.47	100.49	9.50-13.49	83.34-105.79
		3-<18 years	526	28.61	26.33	12.06	58.47	11.30-13.14	54.67-64.83
**Fe**	μmol/L	28 days-<3 months	156	13.06	12.48	2.95	25.93	2.82-3.93	24.75-27.74
		3 months-<1 year	371	9.84	9.80	2.21	19.09	2.08-2.42	19.23-19.88
		1-<18 years	1024	12.88	13.18	2.56	23.84	2.47-2.81	23.40-24.58
**FER**	ng/mL	28 days-<6 months	221	161.03	103.92	13.32	553.78	9.38-17.80	489.28-635.05
		6 months-<6 years	505	61.58	51.17	11.71	173.50	9.01-14.21	158.12-183.97
		6-<18 years	Male 208 Female 180	Male 70.00 Female 61.22	Male 64.09 Female 56.38	Male 16.75 Female 11.25	Male 145.47 Female 135.03	Male 12.08-21.67 Female 8.01-13.82	Male 140.15-154.68 Female 131.17-144.44
**TRF**	g/L	28 days-<1 year	195	2.25	2.29	1.31	3.06	1.18-1.43	2.93-3.17
		1-<18 years	150	2.44	2.42	1.60	3.20	1.13-1.95	3.16-3.32
**UIBC**	μmol/L	28 days-<3 months	123	31.99	32.01	12.87	57.34	11.10-14.79	49.95-55.46
		3 months-<1 year	265	45.10	44.81	27.39	69.65	25.01-29.98	66.99-72.21
		1-<18 years	561	43.39	42.97	27.17	60.49	24.69-29.40	58.19-63.39
**TIBC**	μmol/L	28 days-<3 months	123	44.86	44.58	27.35	63.95	23.16-31.65	60.09-66.42
		3 months-<1 year	265	55.07	53.43	40.15	74.81	38.00-40.59	72.14-76.27
		1-<18 years	561	55.88	55.73	39.93	72.14	39.01-41.01	70.88-74.41
**ASO**	IU/mL	28 days-<1 year	517	6.93	5.96	≤17.40		16.59-18.86	
		1-<6 years	395	6.97	4.34	≤30.08		18.09-33.99	
		6-<18 years	419	52.35	22.70	≤158.76		148.71-176.60	

Note: 1) “LL” represented “Lower Limit”, “UL” represented “Upper Limit”, “CI” represented “Confidence Interval”. 2) The TIBC was a calculated parameter. Once serum Fe and UIBC values are determined, TIBC is calculated using a straightforward formula: TIBC = Fe + UIBC. 3) The ALT/AST was a ratio obtained by directly dividing AST by ALT using result of the same sample.

Among those hepatic function analytes, AST and ALT levels peaked between 28 days and 1 year of age, followed by a gradual decline. At the same time, we also calculated the RIs of the AST/ALT ratio, and the results showed a trend of first increasing and then decreasing with age. For ALP, it exhibited a more complex and fluctuating trajectory, with a marked increase before 6 months of age. Thereafter, ALP levels decreased after 6 months of age, then progressively increased from 1 year onward until 15 years of age. For γ-GT, it was markedly higher before 6 months of age but decreased by approximately 78% thereafter, whereas Prealbumin showed a continuous increase with age. TBIL and DBIL were relatively high at first 2 months of age, and decreased after 3 months of age until 1 year old. Regarding renal function biomarkers, serum Crea maintained low concentrations from 28 days to 12 years of age and increased to near-adult levels after 12 years, aligning with the functional maturation of children's kidneys. Urea and UA gradually increased with age, while CysC decreased with age. For cardiovascular markers, LDH, α-HBDH, CK-MB, and MYO showed a notable increase within the first 3 years of age, then gradually decreased to adult levels. Immunoglobulins exhibited more pronounced dynamic changes: the ranges of IgA, IgG, IgM, and IgE were all significantly lower in children at the first year of age, gradually increased with advancing age, and showed greater sensitivity to age. For anemia indicators, FER in the first 6 months presented elevated levels. Other markers, such as Fe, TRF, and TIBC, showed relatively low age sensitivity. In terms of glucose assessment, the methodological comparison between the hexokinase (HK) and glucose oxidase (GOD) methods yielded highly concordant RIs. In addition, AST, ALT, ALP, Crea, UA, Urea, P, Glu, FER showed gender differences after entering puberty.

### 3.3 Cross-studies comparison of biochemical RIs

To demonstrate the applicability of established RIs, several large-scale studies were compared, as shown in Table S7, which listed the results of pediatric biochemical RIs from the landmark multi-center projects PRINCE (Chinese), CALIPER white paper (Canadian), and others reference like Mayo Clinic Laboratories Catalog [19], Tietz Textbook of Laboratory Medicine [20], Bohn's study [21]. A total of 36 biochemical analytes demonstrated substantially consistent RIs with the selected studies, including AST, ALT, ALP, γ-GT, TP, Alb, PA, TBIL, DBIL, Crea, Urea, UA, CysC, TC, LDL-C, HDL-C, Glu-GOD, Glu-HK, Na, K, CL, Ca, Mg, P, CO_2_, IgG, IgM, IgA, IgE, CK, LDH, MYO, Fe, FER, TRF, TIBC. However, there still lied some discrepancies in a few analytes among studies, which warrant careful consideration of analytical and preanalytical variables. For instance, the upper limit (UL) of TG in our cohort (2.54 mmol/L for 28 days-<2 years; 2.08 mmol/L for 2-<10 years) was substantially higher—by nearly 2- to 3-fold—than those reported in the Mayo study (0.85 mmol/L for 2-9 years), the Tietz textbook (1.12-1.27 mmol/L for 0-4 years), though slightly lower than CALIPER data (2.92 mmol/L for infants; 2.23 mmol/L for 1-19 years). Similarly, the UL for ASO increased with age, reaching 158.76 IU/mL in the 6-<18 years cohort; however, this value was approximately 480 IU/mL lower than Mayo (640 IU/mL for 5-17 years). The differences in the remaining analytes were primarily reflected in the age partitioning format and gender distribution. In addition, α-HBDH, CK-MB, and UIBC were not involved in the above studies, the evaluation was mainly based on the ranges of clinical experience. Totally, the present study provided a direct and objective evidence of Chinese pediatric RIs to facilitate the feasibility of cross-regional comparisons.

### 3.4 Age-dependent distribution characteristics of pediatric eGFR

Due to the lack of a universally recognized formula for accurately evaluating pediatric renal function, this study delineated the age-dependent trajectories of eGFR calculated by seven reported equations, including CKD-EPI_Crea-CysC_, Schwartz Bedside, EKFC_Crea-CysC_, CKiD U25_Crea-CysC_, Counahan-Barratt, CAPA, FAS_Crea-CysC_, across a cohort of 806 pediatric samples. Overall, the eGFR estimates exhibited pronounced age-dependent variations, reflecting the physiological maturation of renal function in this population. Across the age spectrum, the developmental trajectories generally demonstrated a characteristic rapid increase during the first 2 years old, followed by a gradual stabilization. However, substantial discrepancies in the magnitude and specific developmental trends were observed among the different formulas.

**Fig. 3:**
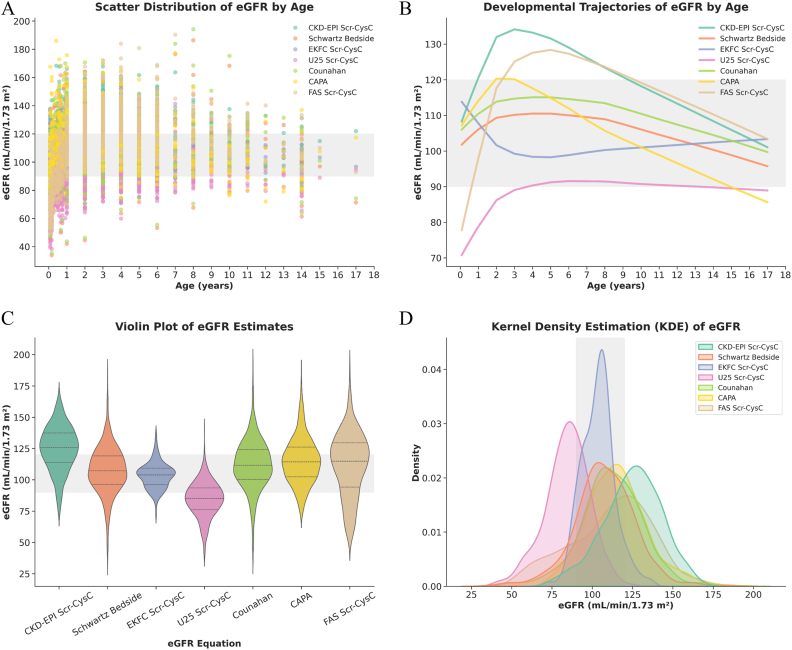
**Age-dependent distribution patterns of pediatric eGFR derived from seven equations**. (A) Scatter plot illustrating the individual eGFR values calculated by seven different formulas across age, with the x-axis set at 1-year intervals to display the comprehensive data distribution. (B) Smoothed developmental trajectories using locally weighted scatterplot smoothing (LOWESS) regression, highlighting the physiological age-related trends of eGFR for each formula. (C) Violin plots displaying the overall distribution, median, and interquartile ranges, demonstrating the systematic inter-equation variability. (D) Kernel density estimation (KDE) curves comparing the probability density distributions of the eGFR outputs among the seven formulas. In all panels, the grey-shaded regions indicate the reference clinical medical decision target range for normal kidney function (90–120 mL/min/1.73 m^2^).

Regarding the age-related trends (Fig. 3A and B), most of the formulas yielded estimates that largely fell within the widely accepted clinical reference range of 90-120 mL/min/1.73 m^2^. The CKD-EPI_Crea-CysC_ equation consistently produced higher eGFR values compared to the other equations in the age of 1 to 9 years old, and those estimates were more frequently located above the hyperfiltration threshold of 120 mL/min/1.73 m^2^, which suggested a tendency toward overestimation. But it gradually decreased to normal levels when entering adolescence. Conversely, the CKiD U25_Crea-CysC_ equation yielded overall estimates that were lower than those of the other formulas. For children under 2 years of age, the FAS_Crea-CysC_ and CKiD U25_Crea-CysC_ estimates were highly sensitive to age fluctuations. Notably, in infants under 1 year, the eGFR values calculated by these two formulas consistently fell below the lower threshold of 90 mL/min/1.73 m^2^. Furthermore, consistent with previous reports indicating its unsuitability for infants, the EKFC_Crea-CysC_ equation demonstrated a tendency to overestimate eGFR in children under 2 years old. Beyond 2 years of age, the EKFC_Crea-CysC_ equation displayed a unique trajectory compared to the others, characterized by a gradual, continuous increase with age. Meanwhile, the Counahan-Barratt and Schwartz Bedside formulas exhibited highly consistent data trends throughout the age spectrum, largely attributable to their structural mathematical similarities. The CAPA estimates presented notable decline with age after 2 years old. Interestingly, during the adolescent phase, estimates from most of the formulas reached closely aligned values.

Analysis of the overall data distributions further highlighted these inter-equation disparities (Fig. 3C and D). The distributions of the EKFC_Crea-CysC_ and Schwartz Bedside equations were the most concentrated, with the bulk of their estimates tightly clustering within the 90-120 mL/min/1.73 m^2^. In contrast, the CKD-EPI_Crea-CysC_ equation exhibited a rightward shift compared with the clinically used reference range. The Counahan-Barratt, CAPA, and FAS_Crea-CysC_ formulas displayed intermediate distributions with a slight leftward shift, whereas the CKD-EPI_Crea-CysC_ equation presented a leftward-shift distribution indicative of potential overestimation. These clues indicated the trajectory of eGFR changes with ages in children under different equations.

## 4 Discussion

The establishment of pediatric RIs represented an urgent and unmet need in clinical practice. Although several multinational initiatives, such as the CALIPER project in Canada, and other cohorts in Australia [22], South Korea [23], Germany [24]and Denmark [25], have reported pediatric RIs, their direct application to disparate populations remained uncertain. Confounding variables, including ethnicity, body mass index, and nutritional status, contribute to significant regional variations, rendering the extrapolation of RIs misleading. Meanwhile, the existing studies have only covered a limited panel of biochemical analytes. To address these critical gaps, this study established comprehensive, age- and gender-specific RIs for 41 biochemical markers in Chinese children. Compared to landmark multi-center projects like CALIPER of Canada and PRINCE of China, our data demonstrated consistent RIs for up to 36 analytes. However, quantified comparisons with other databases revealed notable discrepancies in a few analytes, which warrant careful consideration of analytical and preanalytical variables. For instance, the UL of TG in our cohort was substantially higher by nearly 2- to 3- fold than those reported in the Mayo, Tietz textbook and the Gregory textbook [26], though slightly lower than CALIPER data. These differences likely stemmed from preanalytical such as fasting conditions, dietary intake, population-specific dietary structures during the transition from breastfeeding to solid foods, alongside varying statistical age-partitioning models. Similarly, the UL for ASO increased with age, but the range of 6 to <18 years cohort was far lower than the Mayo's limit. This pronounced variance might reflect differences in the regional prevalence of Group A Streptococcal infections or analytical discrepancies between various immunoassays (e.g., nephelometry versus turbidimetry).

Analysis of data distribution revealed distinct age-dependent dynamics for at least 21 analytes. For pediatric hepatology evaluation, analytical factors when comparing enzymes (e.g., AST, ALT, ALP) across studies should be heavily scrutinized. Variations often arise depending on whether the laboratory employed pyridoxal-5′-phosphate activation or utilized different incubation temperatures. The results of this study showed that AST and ALT levels peaked between 28 days and 1 year of age, followed by a gradual decline. Consistent with the PRINCE and CALIPER studies, sex differences in AST and ALT emerged during adolescence, with AST levels in males being approximately 1.2-fold higher than those in females, which was consistent with the PRINCE and CALIPER findings. For ALT, the male-to-female ratio was 1.6-fold, aligning with the PRINCE study. In contrast, the sex difference in ALT reported by CALIPER was less pronounced (1.1-fold), reflecting potential population-specific variations in developmental sex differences across geographic regions. Bussler et al. [27] also found that serum transaminase levels follow specific age trends, particularly evident during puberty. For ALP, it exhibited a more complex and fluctuating trajectory, with a marked increase before 6 months of age (approximately 1.4-fold higher than in the 6-12 months group). The UL in our study were generally consistent with those of CALIPER and PRINCE. Thereafter, ALP levels gradually decreased after 6 months of age, then progressively increased from 1 year onward until 15 years of age. During adolescence, ALP levels in males were approximately 1.4-fold higher than in females, consistent with the PRINCE study (1.3-fold) but lower than CALIPER (1.8-fold), reflecting differences in skeletal maturation between sexes during this period. After 15 years of age, ALP levels declined markedly in both sexes. However, the limited number of adolescent participants in our study—particularly those older than 15 years—precluded further sex-specific stratification. For reference, the PRINCE and CALIPER studies reported male-to-female ALP ratios of approximately 1.5- to 1.7-fold in this near-adult age groups. Other study of Li et al. also found complex changes of ALP in children [28]. The above distribution characteristics emphasized that when analyzing age-related trends in biochemical analytes, sex-specific differences—particularly during adolescence—should also be considered. Consistent with the PRINCE study, the UL of γ-GT in Chinese children was markedly higher before 6 months of age but decreased by approximately 78% thereafter. In contrast, reports from CALIPER and Mayo indicated that the elevated range of γ-GT persisted until after 1 year of age, reflecting inter-regional differences in the developmental patterns of this analyte across pediatric populations. Prealbumin showed a continuous increase with age, although the RIs established in our study were generally lower than those reported by CALIPER. Regarding bilirubin metabolism, TBIL and DBIL were relatively high at first two months of age due to the immature conjugating capacity of the neonatal liver. As the liver developed, bilirubin decreased after three months of age, and smoothly increased after 1 year old. Compared to CALIPER, this study lacked subgroup for newborns under 28 days old, whose bilirubin levels were relatively higher and more clinically significant. Additionally, our UL of TBIL for the 1-<12 years group was 1.4-1.8 times higher than that of CALIPER, and the 12-<18 years group was very close to the Mayo's range. For DBIL, The UL of 10-<18 years group was highly accordance with CALIPER, However, among individuals under 1 year old, we further identified differences between children aged 3-6 months and those aged 6-12 months, which were not divided in CALIPER. These age-specific variations reflected underlying physiological processes between regions and were essential for ensuring the clinical accuracy of pediatric RIs.

In the context of pediatric renal function analysis, serum Crea maintained low concentrations from 28 days to 12 years of age, and increased to near-adult level after 12 years old, aligning with the functional maturation of infant kidneys. Crea also exhibited an increase after the age of 12-13 in the CALIPER, PRINCE, and Mayo studies. Despite different specific age partitioning between regions, the overall trend of change in results across the studies was consistent. Especially during the puberty stage, Crea levels were slightly higher in males than in females by a fold of 1.2, compared with that of 1.3-fold in PRINCE and Mayo studies, highlighting gender variations in adolescent muscle mass development. Other renal function indicators, such as Urea and UA, also gradually increased with age, especially during adolescence, where gender differences were also evident. Conversely, CysC decreased with age, consistent with CALIPER. With access to paired results of Crea and CysC, and acknowledging the inherent challenges in estimating GFR in children, as the direct measurement of GFR (mGFR) using exogenous clearance markers (e.g., iohexol or inulin) was invasive, labor-intensive, and impractical for routine clinical care, the nephrology community relied almost exclusively on endogenous biomarker-based estimating equations. Thus, we extended our analysis to characterize the age-dependent distribution of eGFR using a panel of established formulas. Although numerous research groups (e.g., CKiD, FAS, EKFC) and pioneers (e.g., Schwartz and Counahan) have developed varied formulas to bridge the gap, the results still performed variously across different age strata due to the maturational changes during childhood [29]. The distributional mapping revealed that while all formulas captured a rapid increase in eGFR during the first 2 years, their absolute values and developmental trajectories diverged significantly. Notably, the EKFC_Crea-CysC_ and Schwartz Bedside equations demonstrated the most concentrated distributions tightly clustering within the clinical range of 90-120 mL/min/1.73 m^2^. A recent study by Hu et al. [15] reporting the direct mGFR in Chinese non-CKD children aged 4.3-9.2 years old established a reference range of 89.3-110.7 mL/min/1.73 m^2^ (median: 100.7 mL/min/1.73 m^2^). In our analysis, within this exact age bracket, the data distribution generated by the EKFC equation (2.5th-95th range for 4-9 years old: 84.0-111.7 mL/min/1.73 m^2^) aligned remarkably closely with Hu et al.’s findings. This strong concordance suggested that the EKFC equation—which utilized a continuous-age concept and population-specific rescaling factors (Q-values)—effectively reflected the renal physiological growth patterns of Chinese pediatric population. In addition, our data highlighted some evaluation biases in several other equations when applied to specific age. The CKD-EPI_Crea-CysC_ equation, primarily calibrated for adults, systematically overestimated renal function in children aged 1 to 9 years, frequently producing values exceeding the hyperfiltration threshold of 120 mL/min/1.73 m^2^, likely due to the application of adult-derived coefficients. Interestingly, its estimates approached normal range when entered adolescence [30]. Meanwhile, the CKiD U25_Crea-CysC_ formula exhibited a pervasive rightward skew, systematically yielding lower estimates compared to other formulas. The CAPA equation, heavily reliant on cystatin C, displayed an anomalous continuous decline in eGFR after 2 years of age. The population under 2 years of age emerged as a particularly vulnerable “grey zone” across some models. Consistent with the original user's suggestion, the EKFC equation demonstrated a clear tendency to overestimate eGFR in children with smaller age. Meanwhile, the FAS_Crea-CysC_ and U25 formulas proved overly sensitive to early age fluctuations, frequently rendering estimates lower than 80 mL/min/1.73 m^2^ in infants under 1 year. This collective instability underscored a limitation that the early nephrogenesis was struggled to perfectly evaluate. Nevertheless, as children progress into adolescence, the estimates from most formulas converged harmoniously, reflecting the stabilization of renal physiology toward adult. These extended findings served as a cross-sectional mapping by delineating the age-specific distributional characteristics of these equations across the most of the age spectrum of Chinese children. It suggested a comprehensive consideration for use of eGFR equation based on their robustness across different ages for pediatric assessments, and urged extreme clinical caution when interpreting formula-derived eGFR under earlier infancy.

Although cardiovascular function undergoes rapid maturation during children, most clinical reference standards for cardiac function biomarkers have been established using adult data, rendering the accurate assessment of myocardial enzyme profiles in children difficult [31]. Our results of myocardial enzymes such as LDH, α-HBDH, CK-MB, and MYO showed notable increase within first 3 years of age, then gradually decreased to adult levels. Particularly for LDH, the total reference ranges in this study were very close to CALIPER and Mayo, but partition showed a small difference. The UL for LDH in this study gradually decreased after 3 years old, whereas others began to decrease after 1 year old. However, α-HBDH, CK-MB, and MYO have not been reported in the aforementioned studies. The findings of our study might therefore contribute to a better clinical understanding of the developmental patterns of cardiac enzyme markers in the pediatric population.

Regarding other parameters, immunoglobulins in children exhibited more pronounced dynamic changes. The ranges of IgA, IgG, IgM, and IgE were all significantly lower in children at first year of age, gradually increased with advancing age, and showed greater sensitivity with age. In this study, each of these immunoglobulins was stratified into six to seven age subgroups, whereas the Mayo employed greater number of subgroups, allowing for more detailed characterization of age-related trends. The reference ranges for IgG and IgM in each age subgroup were generally consistent with those reported by the Mayo. In contrast, IgA levels varied by 2- to 7-fold with increasing age, whereas IgE varied by as much as 6- to 25-fold with age, the ULs of both in the older age were lower than the Mayo values. Collectively, these findings revealed the developmental characteristics of the immune function profile in children and highlighted the need for higher assay sensitivity for immunoglobulin measurements, particularly in younger children. For anemia indicators, the FER in the beginning of six months presented elevated levels, mainly due to the incomplete iron homeostasis regulation mechanism in early stages. Compared with the CALIPER study, the UL in the initial 6 months group in this study was approximately 20% lower. In the 6-<18 years group, the range exhibited sex-specific differences, with ULs approximately twice than those of CALIPER, and were close to the values reported by Tietz. In contrast, the ranges published by Mayo did not incorporate age partitioning. Other markers, such as Fe, TRF, and TIBC, showed relatively low age sensitivity, and our findings were generally consistent with those of both the Mayo and CALIPER studies. In terms of glucose assessment, we found that the established UL was lower than Mayo's range, slightly higher than Tietz's UL for children, but close to the results obtained by Bohn et al. Additionally, we quantified the methodological differences between the HK and GOD methods. Both assays yielded highly concordant RIs, confirming that these specific analytical differences introduce clinically negligible variance in pediatric populations.

In further comparisons with other study, we also noted that Zhong et al. [32] reported ALT results using methods both with and without PLP. The RIs established with PLP were consistently higher than those without in all age groups, and their overall ranges were slightly lower than those established in our study. Notably, the UL for the <1 year age group in our study and the PRINCE study were approximately 1.6-fold higher than those reported by Zhong et al., which might be attributable to the absence of infants under 6 months of age in their study population. For ALP, they reported a consistently complex trend of initial decrease followed by an increase, with sex differences emerging after 10 years of age. Regarding Crea, the RIs were established using both the picric acid (Jaffe) method and the enzymatic method. We found that the Jaffe method yielded higher results than the enzymatic method in children under 3 years of age, whereas after adolescence, the enzymatic method produced higher results than the Jaffe method. Notably, the enzymatic method results were consistent with those of our study. These findings highlighted the need for clinicians to be aware of method-related differences in transaminase and Crea measurement when interpreting results. UA levels increased with age and exhibited sex differences during adolescence, with male values being approximately 1.4-fold higher than female values, and higher than those observed in our study. CysC showed a decreasing trend with age consistent with our findings, though the age-related change was not pronounced. Zhong et al. provided more detailed age partitions for IgG, IgA, and IgM, which all increased with age, and sex-specific partitions were applied during adolescence. Their UL for IgA was 1.3-fold higher than that in our study. For comparison among other regions, several sources of diversity should be carefully considered [33], including preanalytical such as specimen type, analytical factors like platform, reagent composition and calibration traceability, Meanwhile, population characteristics such as ethnicity, geographic location, nutritional status, might affect analyte distributions, particularly during growth and puberty. Moreover, age and sex partitioning strategies and choice of techniques vary across studies. Differences in the number and boundaries of age groups, as well as whether sex-specific intervals were provided, directly affect comparability. Due to the complexity of children's development, ranges of biochemical analytes presented more dynamic changes [34,35]. Several studies have been exploring more appropriate pediatric age partitioning strategies, to minimize reliance on arbitrary or experience-based divisions and, in turn, to narrow the gap between research findings and their direct clinical applicability [36,37].

Partial research groups were attempting to standardize the RIs of universal biochemical analytes through evidence-based methods [[38], [39], [40]], the present research provided more direct and objective evidence of Chinese data to facilitate the feasibility of cross-regional comparisons. However, several limitations of this study should be acknowledged. Firstly, due to practical constraints in recruiting sufficient samples for every specific month or year of age, our initial sample collection was stratified according to predefined physiological developmental stages. However, the final age partitioning for each analyte was primarily determined based on the data distribution characteristics and statistical significance, which might lead to insufficient samples in some partitioning groups. Secondly, due to the limit of sampling, not all the analytes and samples were tested at once. Instead, the testing was conducted in a total of three batches sequentially, with the inclusion of analytes and sample sizes varying across each batch. The comparability of test results for same analyte across batches was ensured through daily quality control and within-batch assessment. Occasionally, insufficient preparation of reagents for some analytes in a timely manner, resulting in the final number of samples available for analysis were relatively limited, such as γ-GT and CO_2_ contained relatively small sample size totally. Thirdly, the sample size of this study was still far lower than that of PRINCE (n = 12352) and CALIPER (n = 9700), especially the number of adolescent participants recruited was limited to meet the requirements, potentially affecting the representativeness and precise estimation of these teenager group when narrower partitioning required. For subgroups with sample size below 40 and skewness was present, as demonstrated by Daly et al. [41], no single method uniformly provided the least biased and most precise estimates for both reference limits, and the robust method could be associated with increased bias and wider confidence intervals. But the bootstrapping was almost as accurate and precise as the transformed parametric method [42]. Another limitation of this study we must acknowledge was that although we evaluated the trajectories of various eGFR equations in children, the absence of directly mGFR limited the strength of conclusions about comparison among equations. Consequently, we could not calculate absolute bias and accuracy to definitively crown one equation as superior. Moreover, this study was only based on single center population, which might introduce selection bias and not be fully representative of the general pediatric population across different geographics. Caution should be exercised when extrapolating these results to other regional populations. Despite these limitations, our study provided comprehensive RIs for a wide panel of biochemical analytes in pediatric population. Our comparative evidence between multiple studies would contribute to the progress of cross-regional harmonized research. Future large-scale, multi-center collaborative studies are warranted to further refine these findings and to facilitate global harmonization initiatives aimed at achieving consistent and standardized interpretation of pediatric RIs across laboratories.

## 5 Conclusion

In summary, this study established comprehensive pediatric RIs for 41 serum biochemical analytes and biochemical profiles in Chinese children based on Mindray chemistry analyzer. Age- and sex-specific partitioning revealed distinct developmental trajectories for 21 analytes. Comparison with multi-center projects demonstrated good agreement for up to 36 analytes, supporting the generalizability of our findings. However, some discrepancies underscored the influence of preanalytical, analytical, and population-specific factors. Additionally, we provided systematic evaluation of specific eGFR equations in children, identifying the EKFC equations as the relatively robust across inter-study comparison. Despite limitations including single-center design and restricted adolescent representation, this study offered valuable RIs for Chinese pediatric populations, facilitated cross-regional harmonization efforts, and informed future study to further refine pediatric laboratory medicine to mitigate the risk of using inappropriate RIs.

## CRediT authorship contribution statement

**Qinlan Liu:** Writing – original draft, Methodology, Formal analysis, Conceptualization. **Xiufa Zhang:** Writing – original draft, Formal analysis, Conceptualization. **Ling Zhuang:** Writing – original draft, Methodology, Conceptualization. **Yidan Wang:** Validation, Investigation, Data curation. **Xian Chen:** Writing – review & editing, Resources, Investigation. **Feng Wang:** Writing – review & editing, Supervision, Project administration, Conceptualization.

## Ethics statement

The study has been performed in accordance with the Declaration of Helsinki and with the approval of the institutional ethics committee of Shenzhen Baoan Women's and Children's Hospital. (Ref: LLSC-2022-03-10-05-KS). The written informed consent was obtained from the minor(s) in addition to parental/guardian consent for the publication of any potentially identifiable images or data included in this article.

## Funding information

Funding was received for the research, authorship, and/or publication of this article. This study was supported by the Zhongnanshan Medical Foundation of Guangdong Province (ZNSXS-20240021) and Wu Jieping Medical Foundation Special Grant for Clinical Research (320.6750.2023-11-43). The funding agency had no role in study conception, data extraction, data analysis, publishing decision or manuscript preparation.

## Declaration of competing interest

The authors declare that they have no known competing financial interests or personal relationships that could have appeared to influence the work reported in this paper.

## Data Availability

Data will be made available on request.
