# Supplementary material for: Establishment of reference intervals for 41 serum biochemical analytes in healthy Chinese children
Source: Pract Lab Med. 2026 May 7;50:e00537. doi: 10.1016/j.plabm.2026.e00537 (PMC13196472; doi:10.1016/j.plabm.2026.e00537)
Supplement: Multimedia component 1 [file mmc1.docx]

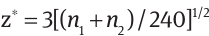

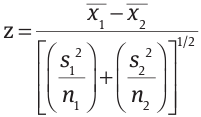


Figure S1. Harris–Boyd equations. Z was Z-calculated and Z* was Z-critical.

Table S1. The approaches and traceability for biochemical analytes in the present study.

| Analyte | Abbreviation | Approaches | Unit | Traceability |
| --- | --- | --- | --- | --- |
| Aspartate aminotransferase | AST | IFCC method (pyridoxal phosphate free) | U/L | WS/T 353-2011 Reference method for determination of catalytic activity concentration of aspartate aminotransferase (pyridoxal phosphate free) |
| Alanine aminotransferase | ALT | IFCC method (pyridoxal phosphate free) | U/L | WS/T 352-2011 Reference method for determination of catalytic activity concentration of alanine aminotransferase (pyridoxal phosphate free) |
| Alkaline phosphatase | ALP | AMP buffer method | U/L | IFCC Reference Measurement Procedure (37℃) for alkaline phosphatase, manual measurement |
| γ-glutamyl-transferase | γ-GT | IFCC method | U/L | IFCC Reference Measurement Procedure (37℃) for γ-glutamyl-transferase, manual measurement |
| Total protein | TP | Biuret method | g/L | Doumas reference method (NIST SRM 927d) |
| Albumin | Alb | Bromocresol green method | g/L | ERM-DA 470K |
| Prealbumin | PA | Immunoturbidimetric method | mg/L | ERM-DA470K |
| Total bilirubin | TBIL | Vanadate oxidation method | μmol/L | Doumas reference method (NIST SRM 916a) |
| Direct bilirubin | DBIL | Vanadate oxidation method | μmol/L | Measurement procedure selected by manufacturer |
| Creatinine | Crea | Sarcosine Oxidase Method | μmol/L | NIST definitive method ID-LC/MS/MS (NIST SRM 914a, NIST SRM 967) |
| Urea | Urea | Urease-glutamate dehydrogenase, UV method | mmol/L | CDC reference method (NIST SRM 912a, SRM 909b) |
| Uric acid | UA | Uricase-peroxidase method | μmol/L | GC-MS |
| Cystatin C | CysC | Latex-immunoturbidimetric method | mg/L | ERM-DA471 |
| Total cholesterol | TC | CHO-POD method | mmol/L | NIST definitive method ID-GC/MS (SRM 911c, SRM 909b, SRM 1951b) |
| Triglycerides | TG | CHO-POD method | mmol/L | NIST definitive method ID-GC/MS/MS (GBW09149, NIST SRM 909b, SRM 1951b) |
| High Density Lipoprotein-Cholesterol | HDL-C | Direct method | mmol/L | CDC beta-quantification (NIST 911c, SRM 1951b) |
| Low Density Lipoprotein-Cholesterol | LDL-C | Direct method | mmol/L | CDC beta-quantification (NIST 911c, SRM 1951b) |
| Creatine kinase | CK | IFCC method | U/L | IFCC Reference Measurement Procedure (37℃) for Creatine kinase, manual measurement |
| Creatine kinase MB isoenzyme | CK-MB | Immuno-inhibition method | U/L | Measurement procedure selected by manufacturer |
| Lactate dehydrogenase | LDH | IFCC method | U/L | IFCC Reference Measurement Procedure (37°C) for lactate dehydrogenase, manual measurement |
| α-Hydroxybutyrate dehydrogenase | α-HBDH | DGKC method | U/L | Measurement procedure selected by manufacturer |
| Myoglobin | MYO | Particle-enhanced immunoturbidimetric assay method | ng/mL | Measurement procedure selected by manufacturer |
| Glucose (GOD) | Glu (G) | Glucose oxidase-peroxidase method | mmol/L | GC-MS |
| Glucose (HK) | Glu (H) | Hexokinase method | mmol/L | NIST definitive method ID-LC/MS/MS, CDC HK method (NIST SRM 965a, SRM 917c) |
| Potassium | K (A&T) | Ion-selective electrode method (indirect method) | mmol/L | SRM 956 |
| Sodium | Na (A&T) | Ion-selective electrode method (indirect method) | mmol/L | SRM 956 |
| Chlorine | Cl (A&T) | Ion-selective electrode method (indirect method) | mmol/L | SRM 956 |
| Calcium | Ca | Arsenazo III method | mmol/L | NIST/CDC/AACC Flame Atomic Absorption method (NIST SRM 956b) |
| Magnesium | Mg | Xylidyl blue method | mmol/L | AAS reference method (SRM 929a) |
| Phosphate | P | Phosphomolybdate method | mmol/L | Measurement procedure selected by manufacturer |
| Carbon dioxide | CO_2_ | Enzymatic method | mmol/L | SRM 351a |
| Antistreptolysin O | ASO | Latex-immunoturbidimetric method | IU/mL | Measurement procedure selected by manufacturer |
| Immunoglobulin G | IgG | Immunoturbidimetric method | g/L | ERM-DA470K |
| Immunoglobulin M | IgM | Immunoturbidimetric method | g/L | ERM-DA470K |
| Immunoglobulin A | IgA | Immunoturbidimetric method | g/L | ERM-DA470K |
| Immunoglobulin E | IgE | Particle-enhanced immunoturbidimetric method | IU/mL | WHO Standard 75/502 |
| Iron | Fe | Colorimetric method | μmol/L | SRM937 |
| Ferritin | FER | Particle-enhanced immunoturbidimetric method | ng/mL | WHO Standard 94/572 |
| Transferrin | TRF | Immunoturbidimetric method | g/L | ERM-DA470k |
| Unsaturated iron-binding capacity | UIBC | Colorimetric method | μmol/L | SRM937 |

Note: As for traceability, biochemical analytes with reference materials are traced to reference materials, and those without reference materials are traced to reference methods.

Table S2. The calibrators and controls for biochemical analytes used in the present study.

| Analyte | Calibrator | Lot. | Control | Lot. |
| --- | --- | --- | --- | --- |
| AST, ALT, ALP, γ-GT, TP, TP, Alb, TBIL, DBIL, Crea, Urea, UA, TC, TG, LDH, α-HBDH, Glu (GOD), Glu (HK), CK, Ca, Mg, P | Multi Sera Calibrator | 150122009 | ClinChem Multi Control (level 1, level 2) | Level 1: 059324009 Level 2: 059424010 |
| PA | Prealbumin Calibrator | 151024001 |  |  |
| HDL-C, LDL-C | Lipids Calibrator | 150424006 |  |  |
| CK-MB | CK-MB Calibrator | 151423007 |  |  |
| K, Na Cl | Ion Selective Electrode Calibrator | 015523024 |  |  |
| ASO | Antistreptolysin "O" Calibrator | 146924005 |  |  |
| IgG, IgM, IgA | Specific Proteins Calibrator | 150723001 |  |  |
| Fe | Fe Calibrator | 145324008 |  |  |
| FER | FER Calibrator | 152724007 |  |  |
| TRF | TRF Calibrator | 152824006 |  |  |
| UIBC | UIBC Calibrator | 153424007 |  |  |
| MYO | MYO Calibrator | 153723003 | Multimmun Control | 058024006 |
| IgE | IgE Calibrator | 153824002 |  |  |
| CO_2_ | CO_2_ Calibrator | 145124017 | CO_2_ Control | 145124017 |
| CysC | Cystatin C Calibrator | 147224005 | Cystatin C Control | 059024003 |

Note: Cell merging indicated that the analytes share the same calibrator or control.

Table S3. Three eGFR formulas used in the present study.

| Formula | Year | Gender | Indicator | eGFR Equation | Supplementary Formula |
| --- | --- | --- | --- | --- | --- |
| CKD-EPI_Crea-CysC_ | 2021 | Male | Crea≤80 μmol/L, CysC≤0.8 mg/L | 135*(Crea/80)^-0.144^*(CysC/0.8)^-0.323^*0.9961^Age^ | / |
|  |  |  | Crea≤80 μmol/L, CysC>0.8 mg/L | 135*(Crea/80)^-0.144^*(CysC/0.8)^-0.778^*0.9961^Age^ |  |
|  |  |  | Crea>80 μmol/L, CysC≤0.8 mg/L  Crea>80 μmol/L, CysC>0.8 mg/L | 135*(Crea/80)^-0.544^*(CysC/0.8)^-0.323^*0.9961^Age^ |  |
|  |  |  |  | 135*(Crea/80)^-0.544^*(CysC/0.8)^-0.778^*0.9961^Age^ |  |
|  |  | Female | Crea≤62 μmol/L, CysC≤0.8 mg/L | 135*(Crea/62)^-0.219^*(CysC/0.8)^-0.323^*0.9961^Age^*0.963 |  |
|  |  |  | Crea≤62 μmol/L, CysC>0.8 mg/L | 135*(Crea/62)^-0.219^*(CysC/0.8)^-0.778^*0.9961^Age^*0.963 |  |
|  |  |  | Crea>62 μmol/L, CysC≤0.8 mg/L  Crea>62 μmol/L, CysC>0.8 mg/L | 135*(Crea/62)^-0.544^*(CysC/0.8)^-0.323^*0.9961^Age^*0.963 |  |
|  |  |  |  | 135*(Crea/62)^-0.544^*(CysC/0.8)^-0.778^*0.9961^Age^*0.963 |  |
| Bedside Schwartz | 2009 | Male/Female | / | 36.5*height/Crea | / |
| EKFC_Crea_ | 2021 | Male | Crea/Q_Cr_<1 | 107.3*(Crea/Q_Cr_)^-0.322^ | ln(Q_Crea_)=3.200+0.259*Age-0.543*ln(Age)-0.00763*Age^3^+0.0000790*Age^3^ |
|  |  |  | Crea/Q_Cr_≥1 | 107.3*(Crea/Q_Cr_)^-1.132^ |  |
|  |  | Female | Crea/Q_Cr_<1 | 107.3*(Crea/Q_Cr_)^-0.322^ | ln(Q_Crea_)=3.080+0.177*Age-0.223*ln(Age)-0.00596*Age^2^-0.0000686*Age^3^ |
|  |  |  | Crea/Q_Cr_≥1 | 107.3*(Crea/Q_Cr_)^-1.132^ |  |
| EKFC_Cysc_ | 2021 | Male/ Female | Crea/Q_cc_<1 | 107.3*(CysC/Q_cc_)^-0.322^ | Q_CysC_=0.83 for <50 years |
|  |  |  | Crea/Q_cc_≥1 | 107.3*(CysC/Q_cc_)^-1.132^ |  |
| EKFC_Crea-CysC_ | 2021 | Male/ Female | / | (eGFR EKFC_Crea_ + eGFR EKFC_CysC_) /2 | / |
| CKiD U25_Crea_ | 2021 | Male/ Female | / | κ*height(m)/Crea | For male, κ is calculated as:  1<12y=39.0*1.008^age-12^,  12<18y=39.0*1.045^age-12^ |
|  |  |  |  |  | For, female, κ is calculated as:  1<12y=36.1*1.008^age-12^,  12<18y=36.1*1.023^age-12^ |
| CKiD U25_CysC_ | 2021 | Male/ Female | / | κ*(1/CysC) | For male, κ is calculated as:  1<15y=87.2*1.011^age-15^,  15<18y =87.2*0.96^age-15^, |
|  |  |  |  |  | For female, κ is calculated as:  1<12y=79.9*1.004^age-12^,  12<18y=79.9*0.97^(age-12^ |
| CKiD U25_Crea-CysC_ | 2021 | Male/ Female | / | (eGFR CKiD U25_Crea_+ eGFR CKiD U25_CysC_)/2 | / |
| FAS_combi_ | 2017 | Male/ Female | / | 107.3/[(α*Crea/Q_Crea_)+(1-α)* CysC/Q_CysC_] | α=Crea/CysC  Q_Crea-male_=0.9mg/dL  Q_Crea-female_=0.7mg/dL  Q_CysC_=0.82mg/L |
| CAPA | 2014 | Male/ Female | / | 130*cysC^-1.069^*age^-0.117^-7 | / |
| Counahan-Barratt | 2021 | Male/ Female | / | 0.43*height/Crea | / |

Table S4. Age-classified sample collected in this study

| Age group | n | % |
| --- | --- | --- |
| 28 days-<6 months | 389 | 14.81% |
| 6 months-<1 year | 319 | 12.14% |
| 1-<3 years | 498 | 18.96% |
| 3-<6 years | 676 | 25.73% |
| 6-<9 years | 313 | 11.91% |
| 9-<12 years | 267 | 10.16% |
| 12-<18 years | 165 | 6.28% |
| Total | 2627 | 100.00% |

Table S5. Sample size and test items for three batches

| Batch | Number of samples | Number of Analytes | Analytes |
| --- | --- | --- | --- |
| 1 | 667 | 28 | AST, ALP, Alb, PA, TBIL, DBIL, Crea, Urea, UA, TC, TG, HDL-C, CK, LDH, α-HBDH, MYO, Ca, Mg, P, IgG, IgM, IgE, IgA, Glu-G, Glu-H, Fe, FER, ASO |
| 2 | 833 | 24，including 4 newly added analytes | PA, TBIL, DBIL, UA, TC, TG, HDL-C, Mg, IgG, IgM, IgE, IgA, CK, LDH, α-HBDH, MYO, FER, TRF, ASO, LDL-C, CK-MB, TRF, UIBC, TIBC |
| 3 | 1127 | 41，including 8 newly added analytes | AST, ALP, Alb, PA, TBIL, DBIL, Crea, Urea, UA, TC, TG, HDL-C, CK, LDH, α-HBDH, MYO, Ca, Mg, P, IgG, IgM, IgE, IgA, Glu-G, Glu-H, Fe, FER, ASO, LDL-C, CK-MB, TRF, UIBC, TIBC, ALT, γ-GT, TP, CysC, Na, K, Cl, CO_2_ |

Note: 1) Underlined analytes were newly added analytes in each batch. 2) The TIBC was a calculated parameter. Once serum Fe and UIBC values are determined, TIBC is calculated using a straightforward formula: TIBC = Fe + UIBC.

Table S6. The original sample size corresponding to each analyte

| AST | ALT | ALP | TP | Alb | PA | TBIL | DBIL |
| --- | --- | --- | --- | --- | --- | --- | --- |
| 1734 | 721 | 853 | 1126 | 846 | 1424 | 1406 | 1425 |
| Crea | Urea | UA | CysC | TC | TG | LDL-C | HDL-C |
| 1441 | 816 | 1650 | 897 | 1594 | 1676 | 1068 | 1644 |
| Glu-H | Glu-G | Na | K | Cl | Ca | Mg | P |
| 818 | 845 | 1113 | 1113 | 1113 | 787 | 1673 | 624 |
| IgG | IgM | IgA | IgE | CK | CK-MB | LDH | α-HBDH |
| 1480 | 1415 | 1289 | 720 | 1436 | 1059 | 1422 | 1635 |
| MYO | Fe | FER | TRF | UIBC | TIBC | ASO | γ-GT |
| 1284 | 1573 | 1139 | 359 | 972 | 972 | 1427 | 196 |
| CO_2_ |  |  |  |  |  |  |  |
| 282 |  |  |  |  |  |  |  |

Note: For γ-GT and CO_2_, we were limited to obtain sufficient reagent, resulting in only 196 and 282 samples were respectively tested.

Table S7. Comparison of clinical chemistry analytes RIs against guidelines and other similar studies.

| Analyte | Present Study | | | | | PRINCE | CALIPER | Mayo | Tietz | Bohn, et al. |
| --- | --- | --- | --- | --- | --- | --- | --- | --- | --- | --- |
|  | Unit | Group | n | LL | UL |  |  |  |  |  |
| AST | U/L | 28 days-<1 year | 261 | 22.29 | 75.70 | 28 days-<1 year：Male 26-73/Female 27-74 1-<2 years：Male 26-55/Female 26-53 2-<13 years：Male 17-40/Female 15-39 13-<20 years：Male 13-33/Female 12-27 | 0-<15 days：32-162 15 days-<1 year：20-67 1-<7 years：21-44 7-<12 years：18-36 12-<19 years：Male 14-35/Female 13-26 | 1-13 years：Male 8-60/Female 8-48 ≥14 years：Male 8-48/Female 8-43 | Male <35/Female <31 | 0-<1 year：25-90 1-<7 years：27-49 7-<13 years：20-40 13-<19 years：Male 17-44/Female 16-28 |
|  |  | 1-<2 years | 120 | 24.48 | 55.68 |  |  |  |  |  |
|  |  | 2-<7 years | 801 | 21.91 | 44.81 |  |  |  |  |  |
|  |  | 7-<13 years | 228 | 13.30 | 38.58 |  |  |  |  |  |
|  |  | 13-<18 years | Male 27 Female 32 | Male 11.21 Female 10.33 | Male 33.44 Female 27.44 |  |  |  |  |  |
| ALT | U/L | 28 days-<1 year | 240 | 8.38 | 70.94 | 28 days-<1 year：Male 10-62/Female 13-80 1-<2 years：Male 11-46/Female 12-47 2-<13 years：Male 8-30/Female 8-27 13-20 years：Male 8-46/Female 6-29 | 0-<1 year：5-33 1-<13 years：9-25 13-<19 years：Male 9-24/Female 8-22 | ≥1 year：Male 7-55/Female 7-45 | Male<45/Female<34 | 0-<1 year：10-49 1-<10 years：10-29 10-<19 years：Male 9-44/Female 8-27 |
|  |  | 1-<7 years | 370 | 8.42 | 30.91 |  |  |  |  |  |
|  |  | 7-<18 years | Male 32  Female 45 | Male 7.15 Female 6.25 | Male 46.55 Female 28.18 |  |  |  |  |  |
| ALP | U/L | 28 days-<6 months | 122 | 135.47 | 517.64 | 28 days-<6 months：119-459 6 months-<1 year：140-362 1-<2 years：150-372 2-<9 years：145-350 9-<12 years：Male 154-431/Female 150-426 12-<14 years：Male 166-526/Female 86-391 14-<15 years：Male 107-520/Female 69-282 15-<17 years：Male 67-382/Female 55-185 17-<20 years：Male 57-174/Female 46-112 | 0-<15 days：90-273 15 days-<1 year：134-518 1-<10 years：156-369 10-<13 years：141-460 13-<15 years：Male 127-517/Female 62-280 15-<17 years：Male 89-365/Female 54-128 17-<19 years：Male 59-164/Female 48-95 | 0-14 days：83-248 15 days-1 years：122-469 1-<10 years：142-335 10 -<13 years：129-417 13-<15 years：Male 116-468/Female 57-254 15-<17 years：Male 82-331/Female 50-117 | 4-15 years：54-369 | 0-<6 months：145-495 6 months-<1 years：155-404 1-<10 years：149-349 10-<12 years：186-440 12-<15 years：Male 202-618/Female 76-419 15-<19 years：Male 59-294/Female 54-143 |
|  |  | 6 months-<1 year | 167 | 118.61 | 361.88 |  |  |  |  |  |
|  |  | 1-<7 years | 344 | 157.74 | 379.32 |  |  |  |  |  |
|  |  | 7-<11 years | 71 | 153.48 | 396.12 |  |  |  |  |  |
|  |  | 11-<15 years | Male 25 Female 30 | Male 122.97 Female 77.39 | Male 530.94 Female 384.43 |  |  |  |  |  |
|  |  | 15-<18 years | 14 | 37.71 | 238.28 |  |  |  |  |  |
| γ-GT | U/L | 28 days-<6 months | 71 | 13.45 | 159.03 | 28 days-<6 months：9-126  6 months-<1 year：6-22  1-<13 years：5-15  13-<20 years：Male 8-35/Female 6-24 | 0-<15 days：23-219  15 days-<1 year：8-127  1-<11 years：6-16  11-<19 years：7-21 | 28 days-<11 months：<178  12 months-<6 years：<21  7-<12 years：<24  13-<17 years：Male <43/Female <26 | Male<55/ Female<38 | / |
|  |  | 6 months-<18 years | 123 | 8.64 | 35.41 |  |  |  |  |  |
| TP | g/L | 28 days-<6 months | 145 | 47.00 | 70.00 | 28 days-<6 months：51-69 6 months-<1 year：57-73 1-<2 years：60-74 2-<6 years：63-77 6-<13 years：67-82 13-<20 years：70-86 | 0-<15 days：53-83 15 days-<1 year：44-71 1-<6 years：61-75 6-<9 years：64-77 9-<19 years：65-81 | ≥1 year：63-79 | Neonates: 46-70 1 week: 44-76 7 months-1 year：51-73 1-2 years：56-75 >2 years：60-80 | / |
|  |  | 6 months-<1 year | 156 | 54.90 | 74.30 |  |  |  |  |  |
|  |  | 1-<6 years | 528 | 59.60 | 75.30 |  |  |  |  |  |
|  |  | 6-<18 years | 234 | 61.90 | 80.70 |  |  |  |  |  |
| Alb | g/L | 28 days-<6 months | 141 | 30.85 | 47.63 | 28 days-<6 months：37-50  6 months-<6 years：43-52  6-<13 years：44-54  13-<20 years：46-56 | 0-<15 days：33-45 15 days-<1 year：28-47 1-<8 years：38-47 8-<15 years：41-48 15-<19 years：Male 41-51/Female 40-49 | ≥12 months：35-50 | 0-4 days：28-44 4 days-14 years：38-54 14-18 years：32-45 | 0-<1 year： 28-48 1-<8 years： 37-47 8-<15 years： 39-49 15-<19 years：Male 41-54/Female 39-50 |
|  |  | 6 months-<18 years | 663 | 39.45 | 49.22 |  |  |  |  |  |
| PA | mg/L | 28 days-<6 months | 288 | 50.40 | 190.53 | / | 0-<15 days：20-120 15 days-<1 year：50-240 1-<5 years：120-230 5-<13 years：140-260 13-<16 years：180-310 16-<19 years：Male 200-350/Female 170-330 | ≤18 years：120-320 >18 years：190-380 | Adult：200-400 | / |
|  |  | 6 months-<8 years | 748 | 78.31 | 233.90 |  |  |  |  |  |
|  |  | 8-<18 years | 372 | 79.99 | 293.46 |  |  |  |  |  |
| TBIL | μmol/L | 28 days-<3 months | 87 | 2.04 | 30.40 | / | 0-<15 days：3.3-283.8 15 days-<1 year：0.8-11.7 1-<9 years：0.8-6.8 9-<12 years：0.8-9.4 12-<15 years：1.7-11.9 15-<19 years：1.7-14.4 | 7-14 days：<256.54 15 days-17 years：≤17.10 | Adult：0-34.21 | 0-<2 year：<3.4-104 2-<12 years：5.1-14 13-<19 years：Male 5.1-29/Female <3.4-12 |
|  |  | 3 months-<1 year | 380 | 1.38 | 9.96 |  |  |  |  |  |
|  |  | 1-<12 years | 698 | 2.42 | 12.75 |  |  |  |  |  |
|  |  | 12 -<18 years | 131 | 3.82 | 17.20 |  |  |  |  |  |
| DBIL | μmol/L | 28 days-<3 months | 136 | 1.30 | 15.63 | / | 0-<15 days：5.7-12.1 15 days-<1 year：0.8-5.2 1-<9 years：0.8-3.4 9-<13 years：0.8-5.0 13-<19 years：Male 1.9-7.1/Female 1.7-6.7 | ≥12 months：0-5.13 | 0-3.42 | 0-<10 year：N/A-<1.71 10-<19 years：Male <1.71-13.7/Female <1.71-10.3 |
|  |  | 3 months-<7 months | 190 | 0.54 | 5.15 |  |  |  |  |  |
|  |  | 7 months-<1 years | 202 | 0.45 | 3.40 |  |  |  |  |  |
|  |  | 1 -<10 years | 570 | 0.73 | 4.52 |  |  |  |  |  |
|  |  | 10 -<18 years | 273 | 1.00 | 7.11 |  |  |  |  |  |
| Crea | μmol/L | 28 days-<2 years | 471 | 16.88 | 34.54 | 28 days-<1 year：Male 17-49/Female 17-52 1-<2 years：Male 18-33/Female 18-32 2-<6 years：Male 23-44/Female 23-44 6-<13 years：Male 34-66/Female 33-61 13-<16 years：Male 45-93/Female 41-75 16-<20 years：Male 60-101/Female 47-76 | 0-<15 days：29-82 15 days-<2 years：9-32 2-<5 years：18-38 5-<12 years：27-54 12-<15 years：40-72 15-<19 years：43-74 | 0-11 months：15.03-37.22 1-5 years：16.80-43.32 6-10 years：22.98-53.92 11-14 years：30.94-76.02 ≥15 years：Male 65.42-119.34/Female 52.16-91.94 | 0-1 year：4-29 2-5 years：4-40 6-9 years：18-46 10 years：19-52 | 0-<2 year：13.3-26.5 2-<5 years：17.7-39.8 5-<10 years：25.6-58.4 10-<15 years：35.4-65.4 15-<19 years：Male 45.1-85.8/Female 43.3-74.3 |
|  |  | 2-<4 years | 290 | 21.74 | 42.19 |  |  |  |  |  |
|  |  | 4-<12 years | 555 | 27.83 | 54.36 |  |  |  |  |  |
|  |  | 12-<18 years | Male 30 Female 33 | Male 39.77 Female 35.24 | Male 93.51 Female 77.70 |  |  |  |  |  |
| Urea | mmol/L | 28 days-<6 months | 133 | 0.99 | 5.32 | 28 days-<6 months：0.8-5.1 6 months-<1 year：1.1-5.7 1-<2 years：2.3-6.5 2-<20 years：Male 2.7-6.8/Female 2.5-6.3 | 0-<15 days：1.0-8.2 15 days-<1 year：1.2-6.0 1-<10 years：3.2-7.9 10-<19 years：2.6-6.8 | 1-17 years：2.50-7.14 | Premature：1.1-8.9 Newborn：1.4-4.3 Children：1.8-6.4 Adult：2.1-7.1 | 0-<1 year：1.4-6.4 1-<10 years：3.2-7.9 10-<19 years：Male 3.9-7.5/Female 3.2-6.4 |
|  |  | 6 months-<1 year | 174 | 1.13 | 5.92 |  |  |  |  |  |
|  |  | 1-<18 years | Male 235 Female 249 | Male 2.85 Female 2.37 | Male 7.03 Female 6.36 |  |  |  |  |  |
| UA | μmol/L | 28 days-<1 year | 580 | 99.91 | 386.35 | / | 0-<15 days：164-757 15 days-<1 year：94-377 1-<10 years：106-289 10-<19 years：Male 156-454/Female 153-349 | Male：1-10 years：142.77-321.24 11 years：160.62-350.98 12 years：184.41-380.73 13 years：202.26-410.47 14 years：220.11-440.21 15 years：237.95-464.01 ≥16 years：220.11-475.91 Female：1 year：124.93-291.49 2 years：124.93-297.44 3 years：130.87-303.39 4 years：136.82-309.34 5 years：136.82-315.29 6 years：136.82-321.24 7-8 years：136.82-327.19 9-10 years：136.82-339.08 11 years：136.82-345.03 12 years：136.82-350.98 ≥13 years：160.52-362.88 | Children：120-320 Adults：Male 210-420/Female 150-350 | 0-<1 year：95-351 1-<12 years：131-333 12-<19 years：Male 172-494/Female 172-404 |
|  |  | 1-<11 years | 777 | 159.96 | 368.38 |  |  |  |  |  |
|  |  | 11-<18 years | Male 101 Female 77 | Male 194.76 Female 123.90 | Male 480.95 Female 395.65 |  |  |  |  |  |
| CysC | mg/L | 28 days-<5 months | 128 | 1.00 | 1.90 | / | 0-<1 month：1.49-2.85 1-<5 months：1.01-1.92 5 months-<1 year：0.75-1.53 1-<2 year：Male 0.77-1.85/Female 0.60-1.20 2-<19 years：0.62-1.11 | / | / | / |
|  |  | 5 months-<1 year | 136 | 0.81 | 1.44 |  |  |  |  |  |
|  |  | 1-<18 years | Male 342 Female 282 | Male 0.72 Female 0.69 | Male 1.18 Female 1.10 |  |  |  |  |  |
| TC | mmol/L | 28 days-<2 years | 658 | 2.30 | 5.58 | / | 0-<15 days：Male 1.10-2.82/Female 1.20-3.23 15 days-<1 year：1.66-6.13 1-<19 years：2.90-5.40 | 2-17 years： Acceptable：<4.4 Borderline High：4.4-5.2 High：≥5.2 | 0-4 years：Male 2.96-5.26/Female 2.90-5.18 5-9 years：Male 3,23-4,89/Female 3.39-5.10 10-14 years：Male 3.21-5.29/Female 3.24-5.31 15-19 years：Male 3.06-4.95/Female 3.08-5.39 | / |
|  |  | 2-<10 years | 664 | 2.63 | 5.78 |  |  |  |  |  |
|  |  | 10-<18 years | 263 | 2.49 | 5.16 |  |  |  |  |  |
| TG | mmol/L | 28 days-<2 years | 681 | 0.44 | 2.54 | / | 0-<15 days：0.93-2.93 15 days-<1 year：0.60-2.92 1<19 years：0.50-2.23 | 2-9 years Acceptable：<0.85 Borderline High：0.85-1.12 High：≥1.13  10-17 years Acceptable：<1.02 Borderline High：1.02-1.46 High：≥1.47 | 0-4 years：Male 0.33-1.12/Female 0.39-1.27 5-9 years：Male 0.32-0.96/Female 0.36-1.43 10-14 years：Male 0.38-1.26/Female 0.44-1.36 15-19 years：Male 0.43-1.62/Female 0.41-1.43 | 0-<19 years：0.5-2.9 |
|  |  | 2-<10 years | 727 | 0.31 | 2.08 |  |  |  |  |  |
|  |  | 10-<18 years | 260 | 0.35 | 1.85 |  |  |  |  |  |
| LDL-C | mmol/L | 28 days-<5 months | 183 | 0.91 | 3.00 | / | / | 2-17 years： Acceptable：<2.8 Borderline High：2.8-3.3 High：≥3.4 | 5-9 years：Male 1.63-3.34/Female 1.76-3.63 10-14 years：Male 1.66-3.44/Female 1.76-3.52 15-19 years：Male 1.61-3.77/Female 1.53-3.55 | 0-<19 years：1.4-4.1 |
|  |  | 5 months-<2 years | 325 | 1.22 | 3.48 |  |  |  |  |  |
|  |  | 2-<11 years | 332 | 1.28 | 3.40 |  |  |  |  |  |
|  |  | 11 -<18 years | 149 | 1.28 | 3.27 |  |  |  |  |  |
| HDL-C | mmol/L | 28 days-<7 months | 274 | 0.40 | 2.30 | / | 0-<15 days：0.40-1.08 15 days-<1 year：0.30-1.85 1-<4 years：0.84-1.63 4-<13 years：0.92-1.88 13-<19 years：Male 0.82-1.77/Female 0.83-1.86 | 2-17 years Low HDL：<1.04 Borderline low：1.04-1.17 Acceptable：>1.17 | 5-9 years：Male 0.99-1.94/Female 0.93-1.89 10-14 years：Male 0.96-1.92/Female 0.96-1.82 15-19 years：Male 0.78-1.63/Female 0.91-1.92 | 0-<6 months：0.6-2.1 6 months-<2 years： 0.7-1.7 2-<14 years： 0.9-2.0 14-<19 years：Male 0.8-1.9/Female 0.8-2.0 |
|  |  | 7 months-<2 years | 322 | 0.62 | 1.80 |  |  |  |  |  |
|  |  | 2-<11 years | 729 | 0.80 | 2.21 |  |  |  |  |  |
|  |  | 11-<18 years | 210 | 0.82 | 2.01 |  |  |  |  |  |
| Glu-HK | mmol/L | 28 days-<2 years | 243 | 3.86 | 5.80 | / | / | ≥1 year：3.89-7.78 | Neonates (1 day) ：2.22-3.33 Neonates (> 1 day)：2.78-4.44 Children：3.33-5.55 Adults：4.11-5.89 | 0-<19 years：3.6-5.8 |
|  |  | 2-<5 years | 169 | 3.60 | 5.90 |  |  |  |  |  |
|  |  | 5-<18 years | Male 121 Female 149 | Male 3.55 Female 3.48 | Male 6.00 Female 5.93 |  |  |  |  |  |
| Glu-GOD | mmol/L | 28 days-<2 years | 243 | 3.86 | 5.78 |  |  |  |  |  |
|  |  | 2-<5 years | 169 | 3.63 | 6.02 |  |  |  |  |  |
|  |  | 5-<18 years | Male 126 Female 149 | Male 3.59 Female 3.61 | Male 6.09 Female 5.94 |  |  |  |  |  |
| Na | mmol/L | 28 days-<6 months | 160 | 136.35 | 147.96 | 28 days-<6 months：137-162 6 months-<1 year：136-143 1-<20 years：138-144 | / | ≥1 year：135-145 | Neonates ：133-146 Infants：139-146 Children：138-145 | 0-<19 years：139-146 |
|  |  | 6 months-<1 year | 147 | 136.81 | 146.93 |  |  |  |  |  |
|  |  | 1-<18 years | 788 | 136.40 | 143.86 |  |  |  |  |  |
| K | mmol/L | 28 days-<1 year | 172 | 3.90 | 5.90 | 28 days-<6 months：4.2-6.3 6 months-<1 year：4.3-5.9 1-<2 years：4.2-5.7 2-<3 years：4.0-5.4 3-<16 years：3.8-5.2 16-20 years：3.6-4.9 | / | 1-17 year：3.6-5.2 | Neonates ：3.7-5.9 Infants：4.1-5.3 Children：3.4-4.7 | 0-<1 year：4.27-6.00 1-<14 years：3.87-5.40 14-<19 years：Male 3.90-5.80/Female 3.82-5.49 |
|  |  | 1-<7 years | 535 | 3.80 | 5.20 |  |  |  |  |  |
|  |  | 7-<18 years | 158 | 3.70 | 5.00 |  |  |  |  |  |
| CL | mmol/L | 28 days-<6 months | 308 | 103.00 | 114.00 | 28 days-<6 months：100-116 6 months-<20 years：98-110 | / | ≥1 year：102-112 | Neonates：98-113 | / |
|  |  | 6 months-<18 years | 788 | 102.00 | 111.00 |  |  |  |  |  |
| Ca | mmol/L | 28 days-<2 years | 248 | 2.34 | 2.73 | 28 days-<6 months：2.2-2.8 6 months-<1 year：2.4-2.8 1-<4 years：2.4-2.7 4-<20 years：2.3-2.6 | 0-<1 year：2.13-2.74 1-<19 years：2.29-2.63 | <1 years：2.17-2.74 1-17 years：2.32-2.64 | Adults：2.15-2.57 | 0-<1 year：2.20-2.84 1-<14 years：2.25-2.69 14-<19 years：Male 2.30-2.77/Female 2.20-2.62 |
|  |  | 2 -<5 years | 160 | 2.39 | 2.68 |  |  |  |  |  |
|  |  | 5-<18 years | 249 | 2.36 | 2.63 |  |  |  |  |  |
| Mg | mmol/L | 28 days-<4 months | 132 | 0.80 | 1.07 | / | 0-<15 days：0.82-1.62 15 days-<1 year：0.81-1.27 1-<19 years：0.86-1.17 | 0-2 years：0.66-1.11 3-5 years：0.66-1.07 6-8 years：0.66-1.03 9-11 years：0.66-0.99 12-17 years：0.66-0.95 | Newborn/2-4 days：0.62-0.90 5 months-6 years：0.70-0.95 6-12 years：0.70-0.86 >12 years：0.66-1.07 | 0-<1 year：0.77-1.05 1-<19 years：0.69-0.92 |
|  |  | 4 months-<2 years | 306 | 0.80 | 1.10 |  |  |  |  |  |
|  |  | 2-<18 years | 856 | 0.80 | 1.00 |  |  |  |  |  |
| P | mmol/L | 28 days-<6 months | 98 | 1.48 | 2.48 | 28 days-<6 months：1.66-2.47 6 months-<1 year：1.54-2.16 1-<2 years：1.48-2.09 2-<6 years：1.43-1.95 6-<12 years：1.31-1.89 12-<15 years：Male 1.21-1.91/Female 1.09-1.82 15-<20 years：Male 0.90-1.67/Female 0.99-1.57 | 0-<15 days：1.80-3.40 15 days-<1 year：1.54-2.72 1-<5 years：1.38-2.19 5-<13 years：1.33-1.92 13-<16：Male 1.14-1.99/Female 1.02-1.79 16-<19 years：0.95-1.62 | 1-4 years：1.39-1.74 5-13 years：Male 1.19-1.74/Female 1.29-1.68 14-15 years：Male 1.13-1.71/Female 1.13-1.58 16-17 years：1.00-1.52 | Children：1.29-2.26 | / |
|  |  | 6 months-<1 year | 74 | 1.39 | 2.25 |  |  |  |  |  |
|  |  | 1-<6 years | 281 | 1.31 | 1.96 |  |  |  |  |  |
|  |  | 6-<18 years | Male 58 Female 81 | Male 1.19 Female 1.13 | Male 1.98 Female 1.88 |  |  |  |  |  |
| CO_2_ | mmol/L | 28 days-<2 years | 135 | 11.15 | 22.51 | / | 15 days-<1 year：10-24  1-<5 years：14-24  5-<15 years：17-26  15-<19 years：Male 18-28/Female 17-26 | / | / | 0-<1 year：13.8-23.3  1-<5 years：16.6-25.3  5-<15 years：Male 18.1-27.3/Female 17.9-27.7  15-<19 years：Male 20.2-27.1/Female 19.9-31.3 |
|  |  | 2-<18 years | 147 | 15.83 | 23.20 |  |  |  |  |  |
| IgG | g/L | 28 days-<6 months | 253 | 0.80 | 8.36 | / | 0-<15 days：3.2-14.0 15 days-<1 year：1.1-7.0 1-<4 years：3.2-11.5 4-<10 years：5.4-13.6 10-<19 years：6.6-15.3 | 0-<5 months：1.00-3.34 5-<9 months：1.64-5.88 9-<15 months：2.46-9.04 15-<24 months：3.13-11.70 2-<4 years：2.95-11.56 4-<7 years：3.86-14.70 7-<10 years：4.62-16.82 10-<13 years：5.03-17.19 13-<16 years：5.09-15.80 16-<18 years：4.87-13.27 | 4 days：7.0-14.8 Adults：7.0-16.0 | 0-<1 year：1.36-8.90 1-<4 years：4.43-12.4 4-<10 years：5.66-13.6 10-<19 years：6.84-15.8 |
|  |  | 6 months-<1 year | 215 | 2.26 | 9.97 |  |  |  |  |  |
|  |  | 1-<2 years | 120 | 3.91 | 11.98 |  |  |  |  |  |
|  |  | 2-<5 years | 308 | 5.10 | 13.20 |  |  |  |  |  |
|  |  | 5-<8 years | 259 | 6.20 | 14.40 |  |  |  |  |  |
|  |  | 8-<11 years | 141 | 6.22 | 15.00 |  |  |  |  |  |
|  |  | 11-<18 years | 146 | 7.94 | 16.76 |  |  |  |  |  |
| IgM | g/L | 28 days-<4 months | 142 | 0.10 | 1.40 | / | 0-<15 days：0.1-0.4 15 days-<13 weeks：0.1-0.7 13 weeks-<1 year：0.2-0.9 1-<19 years：Male 0.4-1.5/Female 0.5-1.9 | 0-<5 months：0.26-1.22 5-<9 months：0.32-1.32 9-<15 months：0.40-1.43 15-<24 months：0.46-1.52 2-<4 years：0.37-1.84 4-<7 years：0.37-2.24 7-<10 years：0.38-2.51 10-<13 years：0.41-2.55 13-<16 years：0.45-2.44 16-<18 years：0.49-2.01 ≥18 years：0.37-2.86 | Adults：0.4-2.3 | 0-<1 year：0.16-1.14 1-<19 years：Male 0.39-1.66/Female 0.48-2.06 |
|  |  | 4-<10 months | 194 | 0.30 | 1.30 |  |  |  |  |  |
|  |  | 10 months-<2 years | 153 | 0.49 | 2.20 |  |  |  |  |  |
|  |  | 2-<5 years | 202 | 0.60 | 2.40 |  |  |  |  |  |
|  |  | 5-<11 years | 345 | 0.60 | 2.50 |  |  |  |  |  |
|  |  | 11-<18 years | 201 | 0.64 | 2.22 |  |  |  |  |  |
| IgA | g/L | 28 days-<4 months | 156 | 0.01 | 0.43 | / | 0-<1 year：0.0-0.3 1-<3 years：0.0-0.9 3-<6 years：0.3-1.5 6-<14 years：0.5-2.2 14-<19 years：0.5-2.9 | 0-<5 months：0.07-0.37 5-<9 months：0.16-0.50 9-<15 months：0.27-0.66 15-<24 months：0.36-0.79 2-<4 years：0.27-2.46 4-<7 years：0.29-2.56 7-<10 years：0.34-2.74 10-<13 years：0.42-2.95 13-<16 years：0.52-3.19 16-<18 years：0.60-3.37 | 4 days：0.00-0.02 Adults：0.7-4.0 | 0-<2 year：<0.33-1.70 2-<6 years：0.37-1.78 6-<14 years：0.58-2.51 14-<19 years：0.71-3.35 |
|  |  | 4-<10 months | 202 | 0.09 | 0.87 |  |  |  |  |  |
|  |  | 10 months-<2 years | 132 | 0.14 | 1.14 |  |  |  |  |  |
|  |  | 2-<5 years | 307 | 0.16 | 1.86 |  |  |  |  |  |
|  |  | 5-<8 years | 191 | 0.50 | 2.38 |  |  |  |  |  |
|  |  | 8-<11 years | 94 | 0.44 | 2.64 |  |  |  |  |  |
|  |  | 11-<18 years | 121 | 0.75 | 2.70 |  |  |  |  |  |
| IgE | IU/mL | 28 days-<5 months | 155 | ≤16.38 | | / | 0-<7 years: <25-440  7-19 years: <25-450 | 0-5 months：≤13 6-11 months：≤34 1-2 years：≤97 3 years：≤199 4-6 years：≤307 7-8 years：≤403 9-12 years：≤696 13-15 years：≤629 16-17 years：≤537 ≥18 year：≤214 | Adults：0-380 | / |
|  |  | 5 months-<2 years | 193 | ≤96.07 | |  |  |  |  |  |
|  |  | 2-<5 years | 46 | ≤147.67 | |  |  |  |  |  |
|  |  | 5-<10 years | 62 | ≤312.49 | |  |  |  |  |  |
|  |  | 10-<14 years | 142 | ≤535.38 | |  |  |  |  |  |
|  |  | 14-<18 years | 20 | ≤409.60 | |  |  |  |  |  |
| CK | U/L | 28 days-<4 months | 138 | 26.20 | 235.39 | / | / | >3 months：Male 39-308/Female 26-192 | Male 46-171/Female 34-145 | 0-<14 year：58-312 14-<19 years：Male 88-903/Female 56-541 |
|  |  | 4 months-<2 years | 328 | 41.13 | 248.37 |  |  |  |  |  |
|  |  | 2-<18 years | 705 | 17.86 | 227.84 |  |  |  |  |  |
| CK-MB | U/L | 28 days-<3 years | 588 | 12.56 | 43.03 | / | / | / | / | / |
|  |  | 3-<18 years | 337 | 8.64 | 25.02 |  |  |  |  |  |
| LDH | U/L | 28 days-<3 years | 797 | 196.16 | 468.09 | / | 0-<15 days：309-1222 15 days-<1 year：163-452 1-<10 years：192-321 10-<15 years：Male 170-283/Female 157-272 15-<19 years：130-250 | 1-11 months：180-435  1-3 years：160-370  4-6 years：145-345  7-9 years：143-290  10-12 years：120-293  13-15 years：110-283  16-17 years：105-233 | 12 months-12 years：180-360 12-60 years：125-220 | 0-<1 year：228-438 1-<12 years：207-383 12-<19 years：Male 136-293/Female 146-279 |
|  |  | 3-<8 years | 247 | 193.49 | 335.66 |  |  |  |  |  |
|  |  | 8-<12 years | 216 | 144.68 | 294.75 |  |  |  |  |  |
|  |  | 12-<18 years | 123 | 131.57 | 241.08 |  |  |  |  |  |
| α-HBDH | U/L | 28 days-<2 years | 627 | 159.24 | 324.44 | / | / | / | / | / |
|  |  | 2-<8 years | 531 | 163.57 | 282.72 |  |  |  |  |  |
|  |  | 8-<12 years | 209 | 121.17 | 225.20 |  |  |  |  |  |
|  |  | 12-<18 years | 120 | 100.64 | 180.80 |  |  |  |  |  |
| MYO | ng/mL | 28 days-<6 months | 192 | 9.58 | 69.41 | / | / | ≤90 | / | / |
|  |  | 6 months-<1 year | 128 | 10.53 | 111.99 |  |  |  |  |  |
|  |  | 1-<3 years | 204 | 12.47 | 100.49 |  |  |  |  |  |
|  |  | 3-<18 years | 526 | 12.06 | 58.47 |  |  |  |  |  |
| Fe | μmol/L | 28 days-<3 months | 156 | 2.95 | 25.93 | / | 0-<14 years：2.8-22.9 14-<19 years：Male 5.5-30.0/Female 3.5-29.0 | Male 8.95-26.86/Female 6.27-25.96 | Adults：5.83-54.50 | 0-<1 year：2.7-21.3 1-<14 years：2.9-29.5 14-<19 years：Male 7.7-34.2/Female 4.1-29.4 |
|  |  | 3 months-<1 year | 371 | 2.21 | 19.09 |  |  |  |  |  |
|  |  | 1-<18 years | 1024 | 2.56 | 23.84 |  |  |  |  |  |
| FER | ng/mL | 28 days-<6 months | 221 | 13.32 | 553.78 | / | 4-<15 days：100-717 15 days-<6 months：14-647 6 months-<1 year：8-182 1-<5 years：5-100 5-<14 years：14-79 Female 14-<19 years：6-67 Male 14-<16 years：13-83 16-<19 years：11-172 | Male 24-336/Female 11-307 | Newborn：25-200 1 month：200-600 2-5 months：50-200 6 months-15 years：7-140 |  |
|  |  | 6 months-<6 years | 505 | 11.71 | 173.50 |  |  |  |  |  |
|  |  | 6-<18 years | Male 208 Female 180 | Male 16.75 Female 11.25 | Male 145.47 Female 135.03 |  |  |  |  |  |
| TRF | g/L | 28 days-<1 year | 195 | 1.31 | 3.06 | / | 0-<9 weeks：1.04-2.24 9 weeks-<1 year：1.07-3.24 1-<19 years：2.20-3.37 | 2.00-3.60 | Newborn：1.17-2.50 | / |
|  |  | 1-<18 years | 150 | 1.60 | 3.20 |  |  |  |  |  |
| UIBC | μmol/L | 28 days-<3 months | 123 | 12.87 | 57.34 | / | / | / | / | / |
|  |  | 3 months-<1 year | 265 | 27.39 | 69.65 |  |  |  |  |  |
|  |  | 1-<18 years | 561 | 27.17 | 60.49 |  |  |  |  |  |
| TIBC | μmol/L | 28 days-<3 months | 123 | 27.35 | 63.95 | / | / | 44.77-71.63 | / | / |
|  |  | 3 months-<1 year | 265 | 40.15 | 74.81 |  |  |  |  |  |
|  |  | 1-<18 years | 561 | 39.93 | 72.14 |  |  |  |  |  |
| ASO | IU/mL | 28 days-<1 year | 517 | ≤17.40 | | / | 0-<6 months：0-0 6 months-<1 year：0-30 1-<6 years：0-104 6-<19 years：0-331 | <5 years：≤70 5-17 years：≤640 | / | / |
|  |  | 1-<6 years | 395 | ≤30.08 | |  |  |  |  |  |
|  |  | 6-<18 years | 419 | ≤158.76 | |  |  |  |  |  |
